# Tamping Ramping: Algorithmic, Implementational, and Computational Explanations of Phasic Dopamine Signals in the Accumbens

**DOI:** 10.1371/journal.pcbi.1004622

**Published:** 2015-12-23

**Authors:** Kevin Lloyd, Peter Dayan

**Affiliations:** Gatsby Computational Neuroscience Unit, London, United Kingdom; Indiana University, UNITED STATES

## Abstract

Substantial evidence suggests that the phasic activity of dopamine neurons represents reinforcement learning’s temporal difference prediction error. However, recent reports of ramp-like increases in dopamine concentration in the striatum when animals are about to act, or are about to reach rewards, appear to pose a challenge to established thinking. This is because the implied activity is persistently predictable by preceding stimuli, and so cannot arise as this sort of prediction error. Here, we explore three possible accounts of such ramping signals: (a) the resolution of uncertainty about the timing of action; (b) the direct influence of dopamine over mechanisms associated with making choices; and (c) a new model of discounted vigour. Collectively, these suggest that dopamine ramps may be explained, with only minor disturbance, by standard theoretical ideas, though urgent questions remain regarding their proximal cause. We suggest experimental approaches to disentangling which of the proposed mechanisms are responsible for dopamine ramps.

## Author Summary

Dopamine has long been implicated in reward-motivated behaviour. Theory and experiments suggest that activity of dopamine-containing neurons resembles a temporally-sophisticated prediction error used to learn expectations of future reward. This account would appear to be inconsistent with recent observations of ‘ramps’, i.e., gradual increases in extracellular dopamine concentration prior to the execution of actions or the acquisition of rewards. We explore three different possible explanations of such ramping signals as arising: (a) when subjects experience uncertainty about when actions will be executed; (b) when dopamine itself influences the timecourse of choice; and (c) under a new model in which ‘quasi-tonic’ dopamine signals arise through a form of temporal discounting. We thereby show that dopamine ramps can be integrated with current theories, and also suggest experiments to clarify which mechanisms are involved.

## Introduction

**Fig 1:**
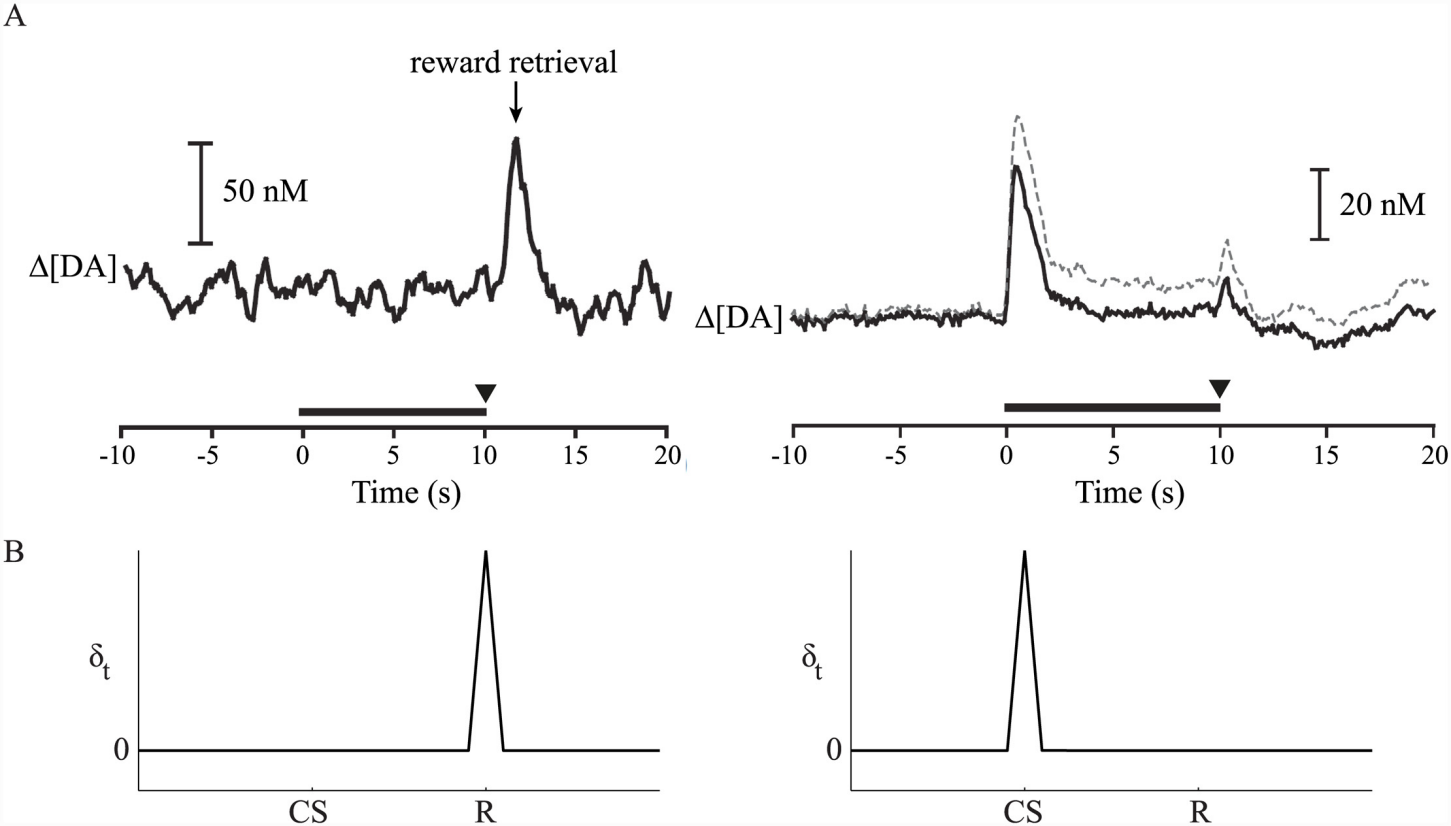
Phasic dopamine signals resemble a temporal difference error. (A) Changes in extracellular dopamine concentration (Δ[DA]) in the nucleus accumbens (NAc) core before (left; single trial) and after (right; mean + SEM) experience of repeated pairings between a predictive cue (horizontal black bar) and a reward (inverted black triangle) delivered at cue offset. Initially, a phasic increase in dopamine is observed at the time of reward delivery. After repeated experience of the relationship between cue and reward, a phasic increase is observed at the time of cue onset, but not at the time of reward, which is still delivered. Adapted from [6], with permission. (B) Models based on temporal difference (TD) learning predict transfer of the TD error *δ*_*t*_ from the time of reward (‘R’; left) to time of predictive cue (‘CS’; right) over the course of learning for both trace and delay conditioning.

Ideas from the field of reinforcement learning (RL) have played an important role in neuroscientific theories of how animals choose actions to gain rewards and avoid punishments. Prominently, it has been suggested [1, 2] that the phasic responses of midbrain dopaminergic neurons resemble a temporal difference (TD) error, a learning signal which facilitates prediction and control of rewarding events [3, 4]. Consistent with this notion, these neurons are activated by unpredicted primary rewards and by cues that predict such rewards, but not by rewards that are themselves reliably predicted. More recent experiments using fast scan cyclic voltammetry (FSCV) to measure rapid changes in extracellular dopamine concentration within projection areas, notably the nucleus accumbens (NAc), find transients which show similar TD-like properties [5–7] (Fig 1).

However, recent reports of ramp-like increases in dopamine concentration preceding self-initiated instrumental responses [8–13] and during approach to spatial locations associated with reward [14] appear to pose a challenge to established thinking. The central issue for TD accounts of dopamine is why such ramping should be observed at all, since TD provides a mechanism for predicting away later dopaminergic activity by earlier—as in the case of the transfer of activity from the time of reward to the time of predictive cues.

One possibility is that these signals have no functional importance, for instance being the result of a process of gated release [14, 15]. In this, a form of ramping activity in the glutamatergic cortico-striatal input might cause the terminals of the dopamine neurons to discharge more of the neuromodulator. This would account for the excess release without any implication for the activity of dopamine neurons—and would mainly pose the question as to how the altered pattern of release could have no effect on striatal activity or plasticity. In the current paper, however, we consider three possible, non-mutually exclusive, functional explanations of NAc dopamine ramps. Firstly, we consider that increases in dopamine which precede an animal’s response may reflect resolution of uncertainty about the time of action. Secondly, we show that ramping may arise if dopamine plays a direct role in modulating the gain of a decision-making process in which value information is integrated over time. Finally, we introduce a discounted model of vigour which may explain the more macroscopic ramping signals observed in [14].

We start by sketching what might be considered the ‘standard’ computational account of dopamine and examining the confounding experimental phenomena.

### Phasic dopamine and TD error

In the main class of TD models of the phasic dopamine response [1, 2, 16], the computational goal of learning is to predict from each state *s* the expected discounted sum *V*(*s*) of the rewards that will be encountered during a trial

$$\begin{array}{c} V\left(s\right)=E\left\{\gamma^{0}r_{t}+\gamma^{1}r_{t+1}+\gamma^{2}r_{t+2}+\ldots |s_{t}=s\right\}, \end{array}$$ (1)

where *r*_*t*_ is the reward delivered at time *t*, and 0 ≤ *γ* ≤ 1 is a discount factor that controls how much weight is given to future relative to immediate rewards. Crucially, the definition of this *state value function* satisfies a (Bellman) consistency condition with respect to each possible next state *s*′:

$$\begin{array}{c} V\left(s\right)=E\{r_{t}+\gamma V\left(s^{\prime }\right)\}. \end{array}$$ (2)

This leads to the idea of using local discrepancies in the value of sampled successive states to drive learning [3, 4, 17, 18]. Thus, the TD error *δ*_*t*_ is defined as

$$\begin{array}{c} \delta_{t}=r_{t}+\gamma V\left(s_{t+1}\right)-V\left(s_{t}\right), \end{array}$$ (3)

and can be used to improve estimates of *V*(*s*). It is exactly this TD error that phasic dopaminergic activity has been hypothesized to represent.

**Fig 2:**
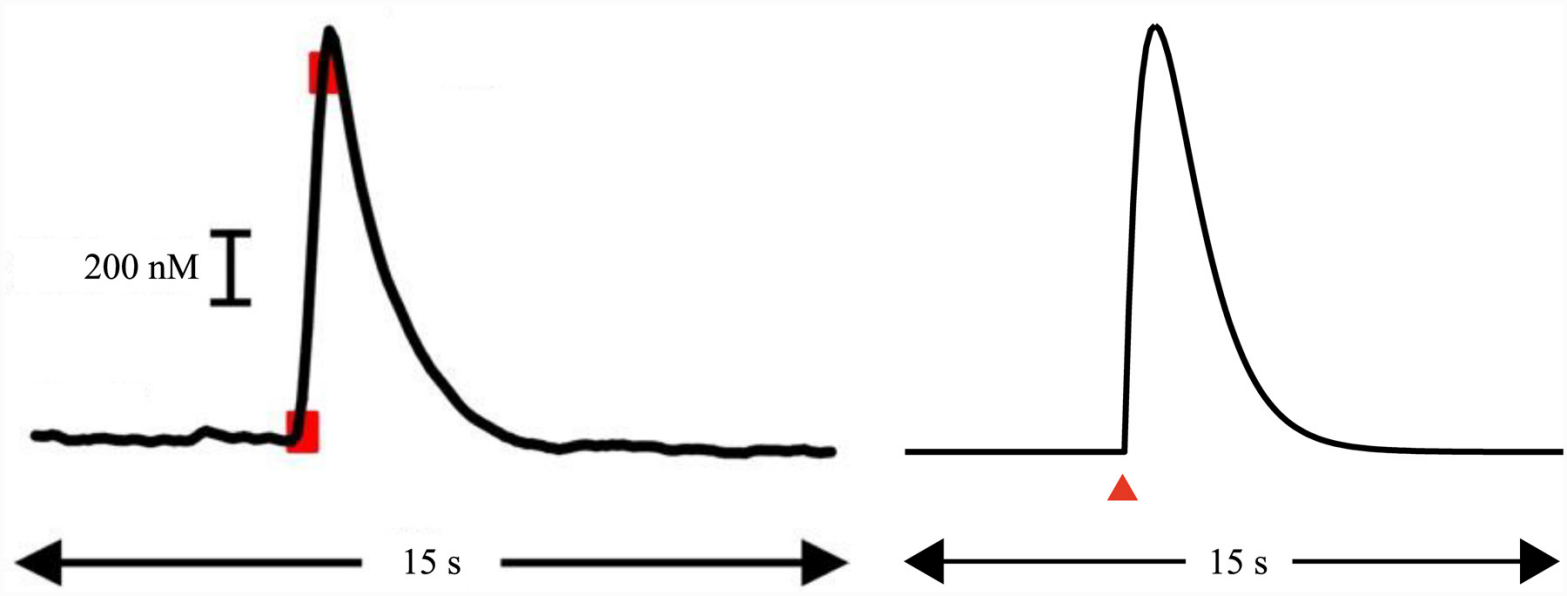
Dopamine response function. Left: Change in NAc extracellular dopamine concentration evoked by electrical stimulation of VTA (red boxes indicate points at which electrical stimulation began and ended). Adapted from [10], with permission. Right: alpha function used to model the effect of a punctate, non-zero TD error (red triangle) on dopamine concentration (Eq (4)).

As noted, in this paper, we consider data on dopamine concentrations in target structures (denoted [DA]) rather than the phasic activity of dopaminergic neurons. These quantities are known to be related [19]; we assume this relationship is simple—a ‘dopamine response function’ (DRF) based qualitatively on the signal evoked in NAc by VTA stimulation (Fig 2). We model the DRF using an alpha function

$$\begin{array}{c} f\left(t\right)=\frac{t}{\xi }e^{1-\frac{t}{\xi }}, \end{array}$$ (4)

with time constant *ξ* = 0.7s set to match experimental observations [10]. In other words, dopaminergic activity at time *t*, which we tendentiously denote $\delta_{t}^{p}$— a phasic TD error —causes an increase in dopamine concentration that peaks after a delay of *ξ* seconds and then decays with time constant *ξ*. Thus, changes in dopamine concentration levels relative to baseline, Δ[DA], are acquired by convolving time-varying activity $\delta_{t}^{p}$ with the DRF described in Eq (4):

$$\begin{array}{c} \Delta \left[\text{DA}\right]\propto \delta^{p}*f\equiv \int_{-\infty }^{+\infty }ds\delta^{p}\left(s\right)f\left(t-s\right). \end{array}$$ (5)

We should note two important caveats to this model. First, there is evidence for richer temporal and non-linear structure in the DRF [20], albeit perhaps most affecting timescales and strengths of responding that are different from those considered here. Of more immediate note is that while there is evidence that fluctuations in dopamine concentration within NAc symmetrically encode positive and negative prediction errors [21], other studies do not show such clear negative deviations from baseline corresponding to a negative prediction error (e.g. [22]). Indeed, evidence suggests that negative prediction errors are represented differently from positive prediction errors in the activity of midbrain dopaminergic neurons: while positive prediction errors appear to correlate positively with the firing rates of dopaminergic neurons, the magnitude of negative prediction errors correlates rather with the duration of a *pause* in burst firing [23, 24], though this itself generates additional complexities. To incorporate the possibility of an asymmetry in how positive and negative prediction errors affect dopamine concentration, below we also examine the effect on dopamine concentration of first asymmetrically scaling negative prediction errors by a factor of *d* = 1/6 [25].

The second caveat is that modulation of striatal dopamine concentrations can occur independently of changes in the observed firing rates of dopaminergic cells. Thus, tonic levels of striatal dopamine are thought to be controlled by the number of active dopaminergic cells rather than by the firing rates of a fixed pool of neurons [26]. Furthermore, a range of mechanisms local to the striatum are known to play a role in regulating dopamine release, including a host of other neurotransmitters such as glutamate, acetylcholine, and GABA (for recent reviews, see [27, 28]).

### Actors and critics

In a case more general than that of learning purely to predict, animals may be allowed to select actions to achieve desired outcomes. A mapping from states to actions is usually referred to as a *policy*, denoted *π*, and the more general problem is to find a policy which maximizes some measure of reward. The TD error signal defined in Eq (3) can be used to evaluate state values with respect to a given policy, *V* ^*π*^(*s*). Given this value function, the agent can potentially improve on its current policy by selecting actions that lead to successor states of higher value. Iteration between successive steps of policy evaluation and policy improvement characterises the *policy iteration* algorithm [29, 30] which is a cornerstone of RL methods [4].

The *actor-critic* algorithm [31], an asynchronous version of policy iteration, is just one of a number of TD-based suggestions for RL [4]. However, it has played a particularly salient role in neural RL modelling [16, 32–34]. In the actor-critic architecture, state values and policy are explicitly represented in different memory structures. The policy structure is known as the *actor*, since it is responsible for selecting actions; and the value structure is known as the *critic*, since it criticizes actions taken by the actor, where this critique takes the form of the TD error described above.

In terms of neural substrate, it has been suggested that the dual learning functions of the actor-critic map to a fundamental division in the functional anatomy of striatum into dorsal and ventral subregions [1, 33, 35, 36]. In particular, the ventral striatum (NAc) is implicated in reward and motivation [37], while the dorsal striatum is implicated in motor and cognitive control [38]. This dissociation is consistent with an implementation of actor and critic components in the dorsal and ventral striatum, respectively [1, 36].

### Tonic dopamine and vigour

Initial theorizing in neural RL focused on tasks involving a simple action or choice between different discrete actions in response to an explicit experimental cue. More recent modelling work has sought to extend standard RL models to other dimensions of choice, thereby making contact with the large experimental literature on free operant tasks in which subjects not only choose between different actions but also when and how quickly to act [39–41].

Two key differences from previous work have been involved in the first collection of models of free operant tasks. Firstly, the agent not only chooses an action *a* to perform, but also an associated latency *τ* with which to perform it. Formally, this entails moving from the usual discrete Markov decision process (MDP) model, in which agent-environment interactions progress at fixed time intervals, to a *semi-Markov decision process* (SMDP) [42], which permits the time spent in a particular state to follow an arbitrary probability distribution. Secondly, rather than assuming that the agent aims—at least approximately—to maximize an expected sum of discounted future rewards, models have assumed an average reward criterion. In this case, the aim is to find a policy that maximizes the long-run average reward rate

ρπ≡limn→∞Eπ1n∑t=0nrt, (6)

which is independent of starting state, assuming ergodicity. The value of a state under policy *π* is now defined relative to the long-run average reward under that policy, *ρ* ^*π*^, and can be denoted ${\tilde{V}}^{\pi }\left(s\right)$ to highlight that this is a *relative* value [4]:

V˜π(s)=Eπ∑k=0∞(rt+k-ρπ)|st=s. (7)

Similarly, the relative action value ${\tilde{Q}}^{\pi }\left(s,a\right)$ of taking action *a* in state *s* is defined as

Q˜π(s,a)=Eπ∑k=0∞(rt+k-ρπ)|st=s,at=a. (8)

**Fig 3:**
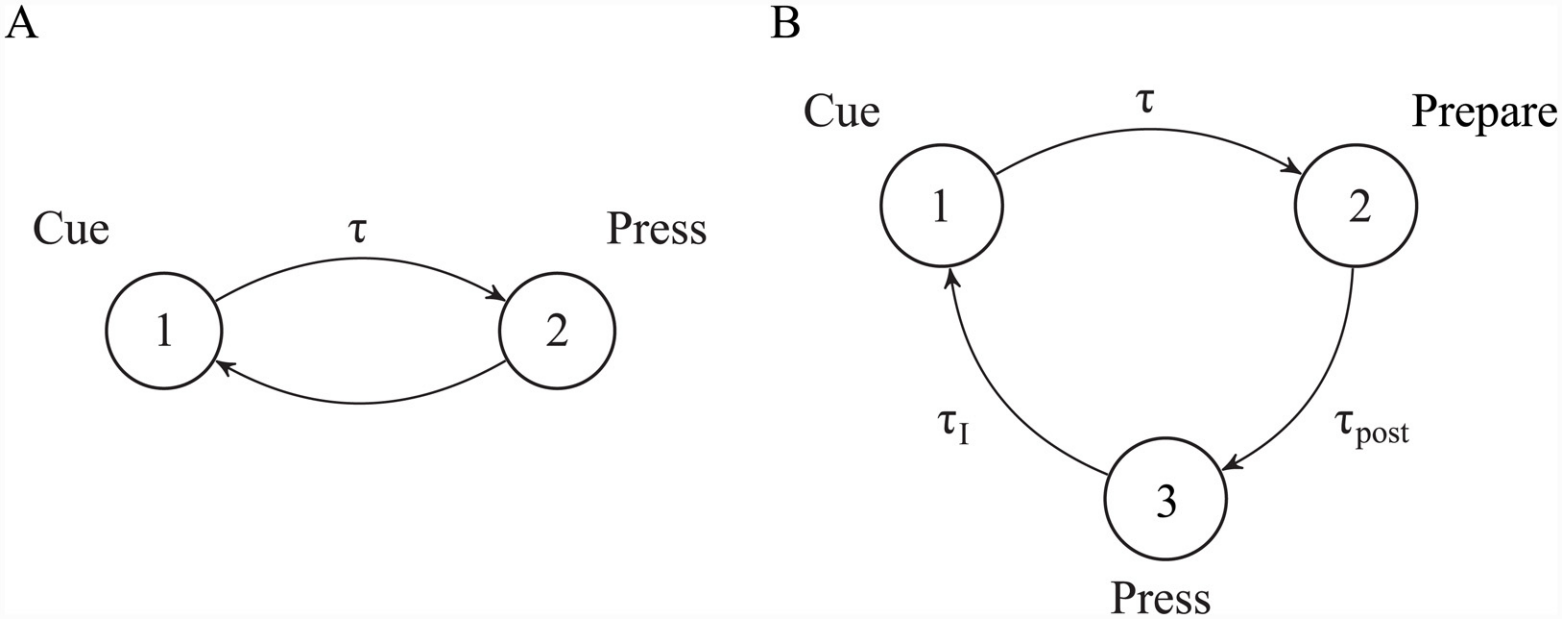
Two conceptions of a cued lever press. (A) A latency *τ* with which to press the lever is selected in an initial cued state (‘1’), leading to completion of the press *τ* seconds later (‘2’). (B) A latency *τ* with which to press the lever is selected in an initial cued state (‘1’), leading to a state of preparedness to press *τ* seconds later (‘2’). Completion of the press (‘3’) occurs only after a subsequent interval *τ*_*post*_. After a further inter-trial interval *τ*_*I*_, the process begins anew.

For example, consider the case in which there is just a single action—a lever press—to perform, and the decision concerns the latency *τ* with which to perform it. For consistency with earlier results, we temporarily consider the case of continuous time. Assume that *τ* is selected, following presentation of an explicit cue, in an initial state ‘1’. After the selected time *τ*, there is a transition to a second state ‘2’ in which the lever press completes and reward is delivered. Subsequent transition back to state 1 follows immediately, and the process begins anew (Fig 3A). Niv et al. [39] considered a hyperbolic cost structure in which a lever press of latency *τ* is more costly depending on its speed. In particular, they adopted the function form for the cost: *a*/*τ* + *b*, where *b* ≤ 0 is a unit cost for the press, and *a* ≤ 0 is a factor which determines the magnitude of hyperbolic dependence on *τ*. Each lever press is assumed to yield an immediate reward of utility *r* > 0. As shown by Niv et al. [39], the theory of average reward RL tells us to select the optimal lever-press latency τ* in state 1 that maximizes the optimal relative Q-value,

τ*=argmaxτQ˜*(1,τ)=argmaxτaτ+b+r-ρ*τ+V˜*(2), (9)

where asterisks are used to indicate values corresponding to an optimal policy. As noted in [39], the optimal latency here is controlled by the opposing forces of the (negative) utility of acting quickly, *a*/*τ*, and the opportunity of cost of acting slowly, −ρ*τ. This latter term arises from Eq (8) since ρ* (which is *ρ* ^*π*^ when executing the optimal policy) is accumulated over all the timesteps comprising latency *τ*. Indeed we have

$$\begin{array}{c} \tau^{*}=\sqrt{\frac{-a}{\rho^{*}}}, \end{array}$$ (10)

which shows that the optimal latency decreases as the average utility rate ρ* increases. Since ρ* also depends on τ*, the problem is recursive, but techniques for finding the optimal solution exist [42, 43].

The connection to current concerns is the proposal that the tonic level of dopamine, especially in NAc, represents the long-run average rate of reward *ρ* ^*π*^, effectively signalling an opportunity cost of sloth [39, 44]. This suggestion is based on a long literature implicating dopamine in the modulation of behavioural vigour [45]. It has been further supported by recent human studies [46, 47], albeit assuming that this long-run average rate arises as a slowly-changing running estimate. The equivalent of the dopamine response function for this signal is unexplored. Dayan [41] has recently broadened the theoretical study of instrumental vigour to include the case of acting to avoid punishment.

### Ramping dopamine concentrations

**Fig 4:**
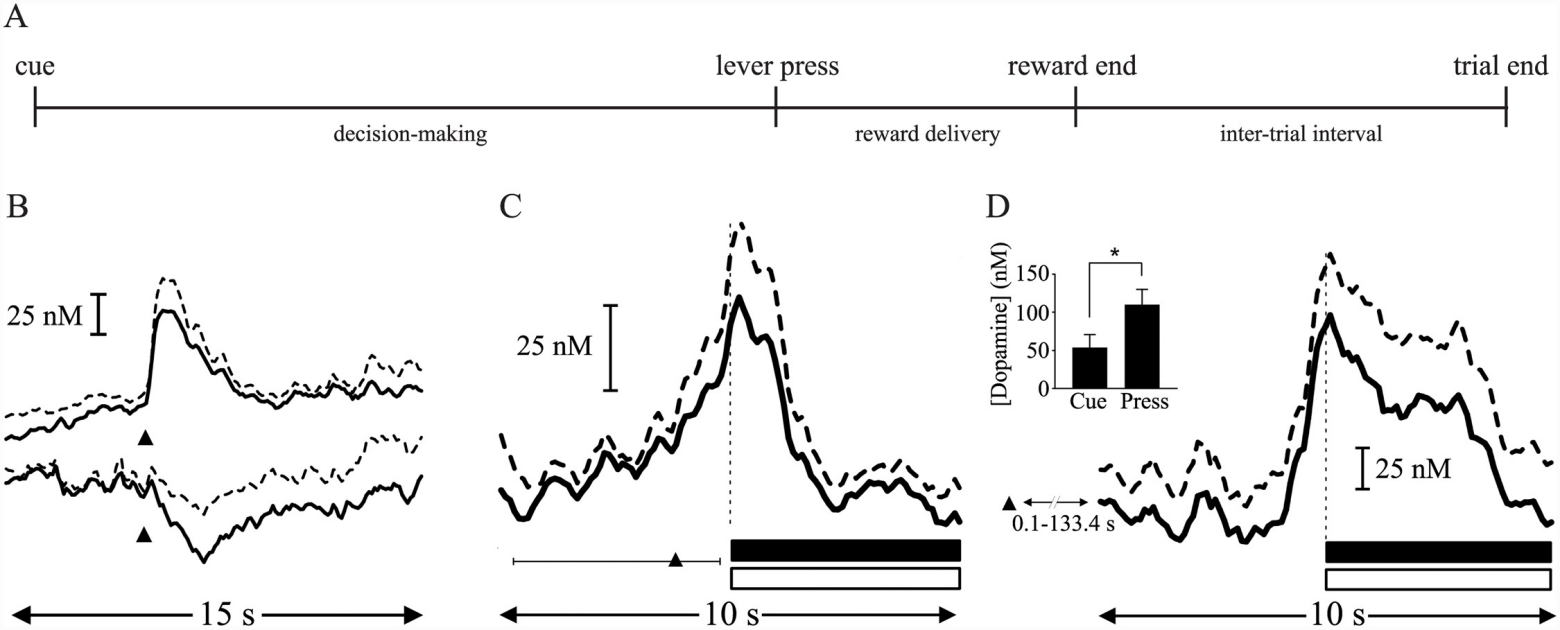
Roitman et al. [10] reported increases in average NAc dopamine concentration that occur shortly before completion of a lever press for reward. (A) Task: rats press a lever at a time of their own choosing for reward (intra-oral sucrose) following a cue indicating that reward is available. (B) Cue presentation (black triangle) evokes a phasic increase in dopamine concentration (mean + SEM) if the cue indicates that reward is available (upper trace), but not when there is no such cue-reward pairing (lower trace); the decrease in signal in the latter case is not caused by dopamine [10]. (C;D) When aligned to time of lever press (vertical dashed line), dopamine concentration is observed to peak at the time of the press, beginning to increase shortly before this time. This is observed both for (C) short-latency trials, where presses are emitted shortly after presentation of the cue (<5 s; average time of presentation indicated by black triangle, range represented by horizontal scale bar) and (D) long-latency trials, where there is a longer delay between cue and response (>5 s). On long-latency trials, average peak dopamine concentration is higher around time of response than around time of cue (D, inset). A lever press leads to both sucrose infusion (black bar) and presentation of a tone-light stimulus (open bar). Figures B–D adapted from [10], with permission.

A first example of the phenomena of interest comes from an experiment by Roitman et al. [10] very similar in structure to the lever pressing case considered above. Following presentation of an explicit cue, a rat could press a lever at a time of its own choosing to receive a sucrose reward (Fig 4A). Cue presentation evoked an increase in dopamine concentration in NAc (Fig 4B, upper trace), but not in control animals for which a lever press did not yield reward (Fig 4B, lower trace). The apparent decrease in signal in the latter case was found not to be caused by a change in dopamine concentration [10]. However, Roitman et al. also observed that, when aligned to the time of lever pressing, average dopamine concentration began to increase a short time before the time of the lever press itself, reaching peak concentration around the time of pressing (Fig 4C and 4D). Crucially, this occurred not only on the majority of the trials (83%) in which animals pressed the lever at relatively short latencies following the initial cue (<5 s; Fig 4C), but also on the smaller number of trials in which animals responded at longer latencies (>5 s; Fig 4D). Similar increases in extracellular dopamine just prior to response have been reported in other FSCV studies [8, 9, 11–13]. Roitman et al. also reported that while cue-aligned and press-aligned peak dopamine concentrations were indistinguishable for short-latency trials (68±19 nM vs. 73±23 nM), press-aligned peak dopamine was significantly larger than cue-aligned peak dopamine on long-latency trials (54±17 nM vs. 110±20 nM; Fig 4D, inset).

**Fig 5:**
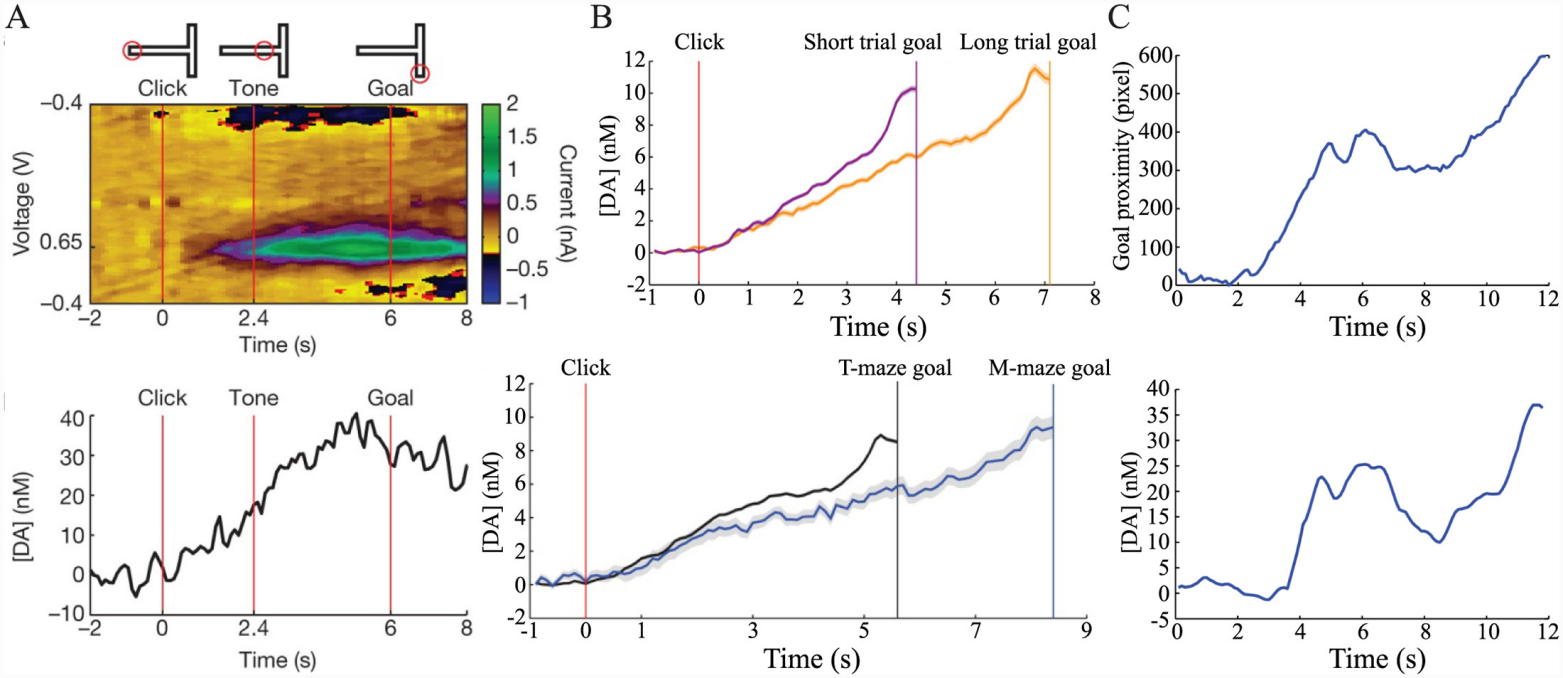
Howe et al. [14] reported gradual increases in striatal dopamine concentration as rats approach reward in a maze. (A) Following an initial warning click, a position-triggered tone indicates to rats which arm of the maze to visit in order to receive reward (upper). Changes in current (middle) and dopamine concentration (lower) measured by FSCV in ventromedial striatum during a single T-maze trial. (B) Average dopamine concentration (±SEM) reaches similar peak values on short vs. long trials for the same maze (upper) and for mazes of different length (lower). (C) Single-trial example showing a close correspondence between the rat’s proximity to the goal (upper) and striatal dopamine concentration (lower). All figures adapted from [14], with permission.

A second, perhaps more dramatic, example of dopamine ramping has recently been reported by Howe et al. [14] (Fig 5). In this study, dopamine concentrations in the striatum were measured using FSCV while rats navigated mazes to obtain remote rewards. It found a gradual increase in dopamine concentration that began at trial onset and ended after reaching the goal (Fig 5A). Whether rats took a relatively short or long time to reach the goal, dopamine peaked at similar concentrations at the goal (Fig 5B, upper). Similarly, dopamine peaked at comparable concentrations at the goal for mazes of different length (Fig 5B, lower). Single-trial examples in which rats paused mid-run showed a remarkable correspondence between proximity to the goal and dopamine concentration (Fig 5C). Furthermore, dopamine ramps scaled with size of reward, so that peak dopamine was higher for larger than smaller rewards (see [14], figure 3).

While we take both of these examples to be instances of dopamine ramping, their explanations may not be identical. Nevertheless, neither case seems to fit neatly with standard RL models because apparently reliable activity is not predicted away by earlier reliable cues.

## Models and Results

We consider three possible, non-mutually exclusive, explanations of NAc dopamine ramps. First, we consider possible sources of predictive uncertainty arising within the actor-critic about when actions will be performed. We show that a TD account in which a prediction error is generated when such uncertainty is resolved just before the action itself may explain pre-response increases in dopamine such as those observed by Roitman et al. [10]. Second, we consider a more direct role for dopamine in decision-making, specifically in setting the gain of a diffusion-to-bound process of value integration. We show that both tonic and phasic fluctuations in dopamine concentration produce what look like average ramping signals in dopamine leading up to the time of decision. Third, we consider the possibility that the prolonged ramping signals observed by Howe et al. [14] may reflect an average reward-like signal that arises within the discounted reward framework. We show that the quasi-tonic signal suggested by our analysis has just the right properties to explain the ramping phenomena observed in [14].

### When will I act? Uncertainty about action timing within the actor-critic

Whether an animal faces a task in which it is free to respond as often and as quickly as it likes, or is limited to a single response within an interval following a cue, it typically has at least some freedom to choose its time of response. In the case of Roitman et al. [10] described above (Fig 4A), rats were free to lever press at a time of their own choosing following a cue marking the start of a new trial. As reported in a number of similar studies, ramp-like increases in NAc dopamine concentration which preceded the time of lever-pressing were observed (Fig 4C and 4D).

From a conventional TD perspective, phasic dopaminergic activity reflects a prediction error. Such errors can be occasioned by changes in latent states associated with the subject’s internal execution of the task, provided that there is some uncertainty associated with these changes. Such uncertainty can be generated by two forms of ignorance: what the critic fails to know about the actor’s choice of when to act, and what both actor and critic fail to know about the passage of time [48, 49].

**Fig 6:**
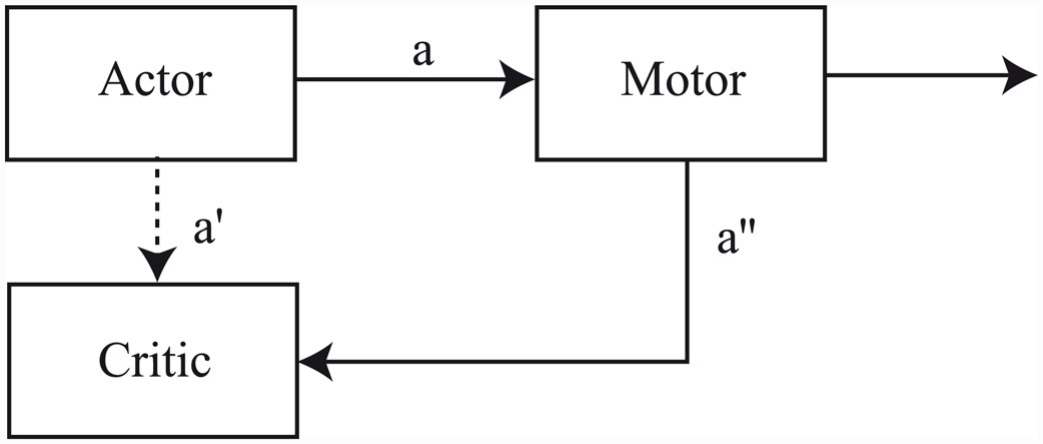
Possible signals received by the critic about action timing. We assume that the actor selects an action *a* (e.g., a latency to lever press) and communicates this choice to downstream pre-motor/motor areas for implementation. We also assume that the critic receives an ‘indirect’ signal *a*′′ via efference copy from downstream areas just prior to performance of the action itself. This latter signal resolves any uncertainty the critic may have about the time of action. The critic may also receive a ‘direct’ signal *a*′ from the actor which carries information about the selected action, and which is received immediately after the actor makes its decision.

Consider first the critic’s knowledge about the temporal decisions of the actor (Fig 6). We assume, reminiscent of studies by Libet and colleagues [50], and consistent with both patterns of cortico-striatal connectivity [51–53] and observed patterns of discharge [54, 55], that internal information proximal to the action, such as some form of motor preparation, is communicated to the critic via efference copy just before it is evident to the experimenter (*a*′′ in Fig 6). This resolves any uncertainty the critic may have about the time of the impending action. The question is what happens at the time that the actor makes its decision about the latency of lever pressing following the initial cue. There are two natural possibilities. One is that the actor *also* intimates its decision about when to act directly to the critic at that time, e.g., via a more indirect form of efference copy (*a*′ in Fig 6) which could be transmitted via interacting cortico-striatal loops or some more direct means [56]. This would then influence the critic’s predictions about future events. The other is that the critic has no such privileged access to the actor’s initial decision, implying that its predictions could be based only on its experience of downstream signals resulting from the actor’s choices.

A second, related issue concerns the realization of timing. If the actor communicates its choice to the critic and the two share the same clock, then there seems to be little room for timing uncertainty to affect the critic’s predictions: regardless of whether the clock is fast, slow, or variable, actor and critic will be in synchrony. On the other hand, if the actor does not specify an exact time of action, or its decisions are subject to additional sources of what the critic will experience as uncontrolled variability (for instance if actor and critic employ different clocks), timing uncertainty may play a role in the critic’s predictions and resulting prediction errors [48, 49].

To explore these issues, we consider the same lever-pressing task described previously (Fig 3A), though with a state space that is augmented to reflect the assumption that the critic may receive internal information about the lever press just before it occurs (Fig 3B). As before, an initial cue (‘1’) is observed, prompting selection of a latency *τ* with which to press the lever. After the selected duration *τ*, which may or may not be known by the critic, the animal transitions to a state of preparedness to press, assumed to be communicated to the critic via efference copy (‘2’; this corresponds to the time at which the critic receives signal *a*′′ in Fig 6). Note that this latter state is distinct from that corresponding to consummation of the lever press itself (‘3’) which is assumed to occur only after a further interval *τ*_*post*_. We set *τ*_*post*_ = 500 ms to correspond roughly with the time with which the so-called ‘readiness potential’ is detected prior to self-initiated action [57, 58]. A reward of utility *r* = 1 is delivered on press completion. Completion of the lever press and reward delivery is followed by a fixed inter-trial interval *τ*_*I*_ = 30 s, after which the process begins anew.

#### Actor

The role of the actor in this scenario is simply to make repeated choices about the latency to lever press. For convenience, we assume that this choice is always made immediately after presentation of the cue. What matters for present purposes is that either through stochastic selection or stochastic execution, there will be a distribution of times that it takes for proximal news of the action to be reported to the critic via efference copy (i.e. the time at which *a*′′ in Fig 6 is transmitted to the critic). We therefore treat this efference copy time as a random variable *T* which, for convenience, we assume to follow a gamma distribution

$$\begin{array}{c} T∼G(k,\theta ), \end{array}$$ (11)

where *k* and *θ* denote shape and scale parameters, respectively.

The source of randomness in *T* interacts with the source of the critic’s information about the lever press. At one extreme of stochastic execution, the latency *τ* could be fixed and variability in *T* is generated by factors not under the actor’s control. We assume the other extreme, in which the distributions of *τ* and *T* are identical, reflecting perfect implementation of the actor’s stochastic choice.

#### Critic

The role of the critic is to learn the relative state values corresponding to the actor’s policy. In the case where the critic only receives indirect information about the actor’s choices, the critic will nevertheless have expectations about *T* based on past experience. Such expectations can be summarized in the form of a ‘prior’ distribution *P*(*T*). If the critic additionally receives direct information about the actor’s choice, the critic can update its beliefs about when engagement will occur based on this information. In the latter case, the critic’s expectations can be summarized as a posterior distribution *P*(*T*|*τ*).

**Fig 7:**
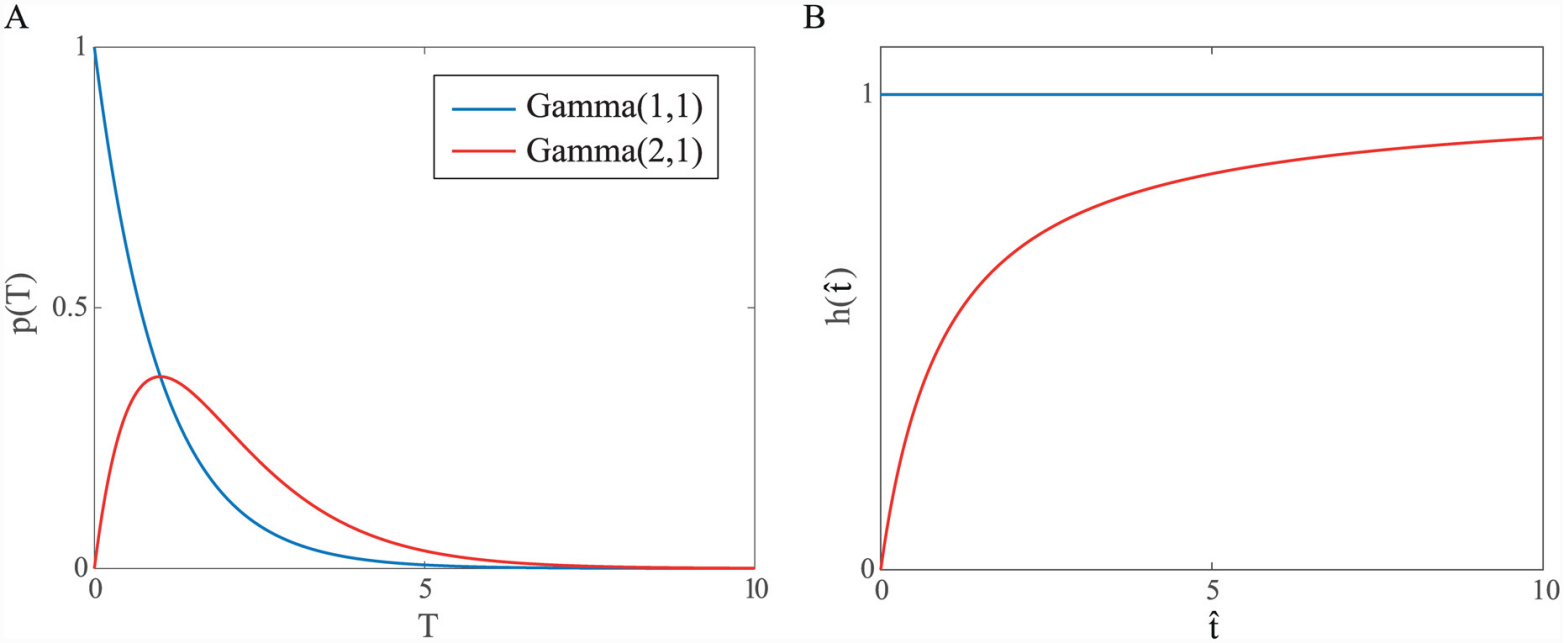
Constant and variable hazard functions. (A) Two different gamma densities of the time *T* at which the critic receives notification of an impending lever press. (B) Corresponding hazard functions $h\left(\hat{t}\right)=\lim_{\Delta \hat{t}\to 0}\left\{P\left(T\leq \hat{t}+\Delta \hat{t}\;|T>\hat{t}\;\right)/\Delta \hat{t}\right\}$. Note that the hazard function is constant in the G(1,1) case, but increases with time in the G(2,1) case.

In either case, how the critic’s beliefs evolve will depend on the passage of time according to the critic’s clock. We denote by $\hat{t}$ the critic’s perceived time since the initial cue is presented. Within state 1, it will then be assumed that the critic estimates the relative values of ‘microstates’ $\left\{1,\hat{t}\right\}$. Whether or not the critic receives direct information about the actor’s choice, at time $\hat{t}$ there are only two possibilities for the immediate future: either the critic receives notice (*a*′′) that the lever press is imminent, $T\leq \hat{t}+\Delta \hat{t}$, or it doesn’t, $T>\hat{t}+\Delta \hat{t}$, where $\Delta \hat{t}$ denotes some short slice of critic time. The probabilities of these events are conditional on $T>\hat{t}$, since it is assumed that the critic has not yet received any such signal. The conditional probability of the critic receiving notice of an imminent press in the immediate future $P(T\leq \hat{t}+\Delta \hat{t}|T>\hat{t})$ is closely related to the *hazard function*. Two general cases are of particular interest and are explored below: T∼G(1,1), in which case the hazard function is constant (i.e. the conditional probability of engagement at any time $\hat{t}$ following the cue is the same), and all other cases—we focus on a G(2,1) distribution for convenience—where the hazard function changes over time (Fig 7).

From these considerations, it is straightforward to write down expressions for the relative values of states $\left\{1,\hat{t}\right\}$. In the case where the critic only receives information about choice indirectly, these values satisfy

$$\begin{array}{cc} {\tilde{V}}^{\pi }\left(\left\{1,\hat{t}\right\}\right)= & P\left(T>\hat{t}+\Delta \hat{t}|T>\hat{t}\right){\tilde{V}}^{\pi }\left(\left\{1,\hat{t}+\Delta \hat{t}\right\}\right)\\ & +P\left(T\leq \hat{t}+\Delta \hat{t}|T>\hat{t}\right){\tilde{V}}^{\pi }\left(2\right)-\rho^{\pi }\Delta \hat{t}, \end{array}$$ (12)

which is a probability-weighted average of the relative values of remaining in state 1 and transitioning to state 2. The case in which the actor directly communicates information about its choice (*a*′) is slightly different due to the possibility of timing uncertainty. Based on the interval-timing literature [59], we assume that the uncertainty about *T* increases with choices of longer latency. For convenience, we consider this conditional distribution to be Gaussian with a standard deviation that scales with *τ*:

$$\begin{array}{c} P(T|\tau )=N(T;\tau ,\sigma =k\tau ), \end{array}$$ (13)

where *k* is the scaling constant. The expression for relative values is identical in form to Eq (12), but these now depend on *τ*:

$$\begin{array}{cc} {\tilde{V}}^{\pi }\left(\left\{1,\hat{t}\right\},\tau \right)= & P\left(T>\hat{t}+\Delta \hat{t}|T>\hat{t},\tau \right){\tilde{V}}^{\pi }\left(\left\{1,\hat{t}+\Delta \hat{t}\right\},\tau \right)\\ & +P\left(T\leq \hat{t}+\Delta \hat{t}|T>\hat{t},\tau \right){\tilde{V}}^{\pi }\left(2\right)-\rho^{\pi }\Delta \hat{t}. \end{array}$$ (14)

In this case, we additionally consider that there is likely some delay between cue onset and the time at which the critic receives information about the actor’s choice. Assuming that the actor makes its decision immediately at cue onset (*t* = 0) and denoting by *ϵ* the delay in intimating this decision to the critic, the relative value of the initial state of ignorance is

$$\begin{array}{c} {\tilde{V}}^{\pi }\left(\left\{1,0\right\}\right)=-\rho^{\pi }\epsilon +\int_{\tau }d\tau \;p\left(\tau \right){\tilde{V}}^{\pi }\left(\left\{1,\epsilon \right\},\tau \right). \end{array}$$ (15)

Whether or not the actor directly reports its choice to the critic, the relative values of states 2 and 3 are straightforward since it is assumed that subsequent state transitions are independent of choice, and their occupation times (*τ*_*post*_, *τ*_*I*_) deterministic. A more general version of the model would also include uncertainty regarding occupation times *τ*_*post*_ and *τ*_*I*_ but we ignore this here since our main focus of interest is on the events occurring between cue and lever press.

#### TD errors and dopamine concentration

Given the critic’s relative state values, we are particularly interested in TD errors and their dopaminergic instantiation. Note that TD errors are inevitable in all cases we consider, either due to the random nature of *T* in the case of indirect communication, or due to timing uncertainty in the case where there is additional direct communication of *τ*. Under the conventional average reward formulation described above, TD errors take the form [60]:

$$\begin{array}{cc} \delta_{t} & =r_{t}+{\tilde{V}}^{\pi }\left(s_{t+1}\right)-{\tilde{V}}^{\pi }\left(s_{t}\right)-\rho^{\pi }\\ & =\delta_{t}^{p}-\rho^{\pi }, \end{array}$$ (16)

where $\delta_{t}^{p}$ is assumed to constitute the phasic component of the error signal reflected in phasic dopaminergic activity, and average reward rate *ρ* ^*π*^ is assumed to be reflected in a constant, tonic level of dopamine. For the moment, we ignore the tonic component of the error term, *ρ* ^*π*^, and only report signals arising from the varying phasic signal $\delta_{t}^{p}$. Changes in dopamine concentration Δ[DA] are therefore modelled by convolving $\delta_{t}^{p}$—either symmetrically or asymmetrically scaled—with the DRF, as per Eq (4).

### Uncertainty resolution, TD errors, and the pre-response dopamine signal

**Fig 8:**
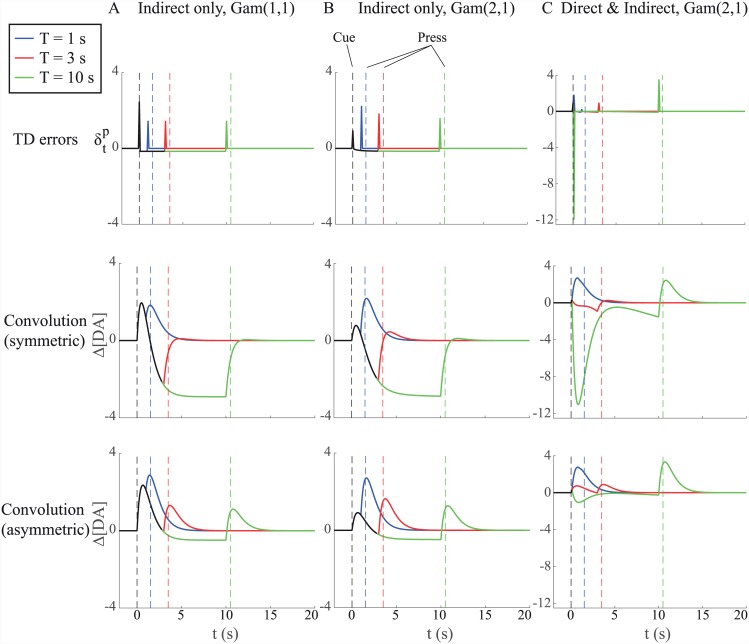
Pattern of prediction errors depends on the nature of communication between actor and critic. In each case (A–C), we consider signals for three particular times of *T* at which the critic receives notice of the impending lever press: 1 s (blue), 3 s (red), and 10 s (green). Parts of the signal where there is overlap between two or more different times of *T* are plotted in black. In each case, we plot TD errors (top), TD errors convolved with symmetric kernel (middle), and TD errors convolved with ‘asymmetric’ kernel (bottom). (A) Indirect communication (*a*′′) only, T∼G(1,1). (B) Indirect communication (*a*′′) only, T∼G(2,1). (C) Both direct and indirect communication (*a*′; *a*′′), T∼G(2,1), with timing uncertainty (uncertainty scaling constant *k* = 0.1). Vertical dashed lines indicate times of observable events, i.e. cue presentation (*t* = 0, black) and lever presses (*t* = *T*+*τ*_*post*_, coloured). Note the difference in *y*-axis scaling between (A;B) and (C). Model parameters: *a* = −1, *b* = 0, *r* = 1, *τ*_*post*_ = 0.5 s, *τ*_*I*_ = 30 s.

Given the models described in the previous section, we consider results from three different cases: two in which the critic only receives information about the lever press indirectly, and one in which the critic additionally receives direct information from the actor. In each case, we consider the effect of the critic receiving notice of impending action at different times—*T* = {1, 3, 10} seconds—on the TD error $\delta_{t}^{p}$, and evaluate the resulting change in dopamine concentration Δ[DA] under both symmetric and asymmetric encoding assumptions. Results for all the cases are summarized together in Fig 8.

#### Indirect communication only

We first consider the case in which the critic receives only indirect information about the actor’s choice. As mentioned above, there are two general cases of interest: where the hazard function is constant (i.e. the conditional probability of engagement at any time $\hat{t}$ following the cue is the same), and where it changes over time.

In the case of a constant hazard function, corresponding to T∼G(1,1), the size of TD error occurring on transition to the state of preparedness does not vary with latency (Fig 8A, upper). This is precisely because the conditional probability of this transition does not vary over time. Note also that after an initial positive TD error, the error signal remains at a constant negative value between the time of cue presentation and the time at which the critic receives efference copy. This constant negative TD error is again a consequence of the flat hazard function. Hazard-related suppression in the spiking activity of dopamine neurons before the occurrence of variably timed (external) reward-related events has been observed in a number of experimental studies [61–63], consistent with reporting of such a negative TD error. The initial positive TD error in response to the cue reflects a positive average value for each trial. Even though the TD error generated in response to the critic’s receiving notice of impending action is constant, differences are observed as to how dopamine concentrations [DA] change for different latencies (Fig 8A, middle and lower). For example, on short-latency trials, TD errors in response to cue onset and receipt of efference copy can combine to produce a larger peak [DA] signal (Fig 8A, lower).

In the G(2,1) case, the hazard rate is not constant. Then, efference-related TD errors decrease with longer latencies (Fig 8B, upper). This is due to the monotonic increase in probability that the lever press will occur with the passage of time—the event is increasingly expected. The decrease in TD error for longer latencies is mirrored in a decrease in the peak [DA] signal (Fig 8B, middle and lower). Interestingly, exactly this pattern of decreasing TD-related activity with time has recently been reported in dopaminergic neurons in response to presentation of a movement trigger signal, where presentation times were randomly drawn from a uniform distribution, while monkeys performed a reaching task [63].

#### Direct and indirect communication

In the case that the critic additionally receives initial information about the actor’s choice of latency, exactly the opposite trend is observed in TD errors occurring just before pressing: they *increase* with latency (Fig 8C, upper). This is due to the assumption that the critic is more uncertain about the time of engagement for longer choices of *τ*. Conversely, TD errors occurring just after the cue, corresponding to the time at which the critic receives initial information about the actor’s choice, *decrease* with *τ*. In particular, choice of a relatively long latency can generate a pronounced negative TD error due to the low relative value of long trials. There is in all cases, however, an initial, brief positive prediction error corresponding to the positive value of the critic’s state of ignorance prior to receiving information about the actor’s choice (c.f. Eq (15)). Again, the pattern in TD errors is mirrored in the resulting [DA] signal, with the decrease in [DA] for a long *τ* being strongly dependent on whether the DRF is assumed to be symmetric or asymmetric (Fig 8C, middle and lower).

**Fig 9:**
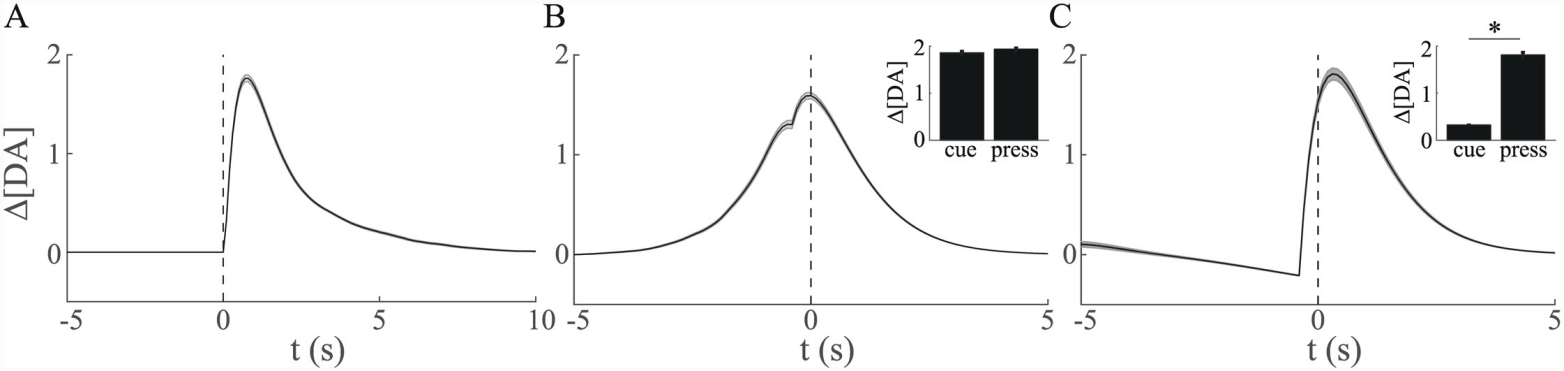
Simulated cue- and press-aligned changes in dopamine concentration, for comparison with Fig 4. Simulated average changes (±SEM) in dopamine concentration for the case where the critic receives both direct and indirect communication, asymmetric convolution of TD errors. (A) Cue-aligned, all trials. (B) Press-aligned, short-latency (<5 s) trials. (C) Press-aligned, long-latency (>5 s) trials. Insets show average peak changes (+SEM) in dopamine around the time of cue presentation and time of lever press. Number of simulated trials *N* = 1000, realizations of *T* drawn G(2,1). Model parameters as before: *a* = −1, *b* = 0, *r* = 1, *k* = 0.1, *τ*_*post*_ = 0.5 s, *τ*_*I*_ = 30 s.

It is this case, especially when positive and negative TD errors are differentially scaled (Fig 8C, lower), that seems to offer the best qualitative fit to the results in [10] that are shown in Fig 4. Fig 9 shows simulated average dopamine concentrations, where the averages are aligned to the various key events in a trial (and separated by latency). Not only do we see a similar signal produced by presentation of the cue (Fig 9A), but we see a qualitative match in press-aligned average signal for short- and long- latency trials (Fig 9B and 9C). Thus, on short-latency trials, we see a pronounced ramping which peaks at the time of the press (Fig 9B). Furthermore, we observe no difference in peak signal when aligned to either cue or press events (Fig 9B, inset). On long-latency trials, just as seen in Roitman et al.’s data, ramping is somewhat less pronounced but similarly begins prior to the press and peaks around the time of press completion (Fig 9C). Furthermore, unlike short-latency trials, the peak [DA] signal is significantly larger around the time of the press than at the time of the cue (Fig 9C, inset).

### A more direct role for dopamine: Setting the gain of value accumulation

Our first possible account of ramping, the TD account of pre-response signals described above, assigns dopamine a passive role in decision-making: increases in dopamine reflect a latent state transition arising from a decision to act which has already been made. However, experimental evidence suggests that accumbens dopamine could also play a more causal role. For example, Phillips et al. [9] found that electrically-evoked dopamine transients in NAc increased the probability that rats would lever press for cocaine immediately afterwards, further commenting that videotaped behavioural records showed that stimulation led to immediate changes in behaviour, notably behavioural sequences up to and including lever approach. Relatedly, Nicola [64] found that blocking dopamine signalling in NAc impaired rats’ ability to approach and press a lever for food, but only when animals were likely to have to re-engage with the task by following a novel sequence of actions to approach the lever. Such findings have led to the suggestion that accumbens dopamine is necessary for ‘flexible approach’ [64]. In fact, models associating phasic dopamine with a TD error signal have long considered a dual role for dopamine in which indirect effects on behaviour, involving learning, are accompanied by direct ones [1, 65–67]. We next explore a second potential mechanism for ramping signals. In particular, we show that a particular decision-making scheme which couples dopamine directly to the decision process also generates dopamine ramps.

A rich vein of work in psychology and neuroscience revolves around the idea that the brain implements some version of the sequential probability ratio test (SPRT), a sometimes optimal procedure for two-alternative forced-choice decisions under uncertainty [68, 69]. While the SPRT and its close associates are usually considered in relation to decision making under state uncertainty, as when there is doubt about whether the overall motion of a random dot field is to the left or right [70], such models have also been applied with some success to memory-based [71] or value-based [72] decisions in which sensory information is absent or unambiguous. We consider the possibility that this arises from accumulation of value information, in which information stored in synapses is read out via spike trains in a temporally extended manner. A prominent realization of the SPRT is the so-called drift-diffusion model (DDM) which we describe in detail below [68, 69, 71, 73–75]. In particular, this can be shown to be a suitable abstraction of a particular sort of neural circuit involving competition between two (or sometimes more) populations of neurons representing the choices [73, 76, 77].

One of the earliest computational suggestions for the role of dopamine and other catecholamines was that by influencing the excitability of neurons [78], they could influence gain control in such circuits, and thereby influence the course of decision-making [79, 80]. Such models were originally conceived of in terms of cortical decision-making circuits; however, for instance, Frank’s [81] neural network model of the basal ganglia assumes that dopamine controls the relative excitability of direct (‘Go’) and indirect (‘Nogo’) pathways via different dopamine receptor subtypes, thereby influencing both the propensity and latency to act. Specifically, higher levels of dopamine shift the balance of activity in favour of the ‘Go’ pathway, leading to a greater propensity to act and faster reaction times. Dopaminergic modulation of excitability in this model can also be interpreted in terms of gain-setting [81].

Here we bring together these two ideas—of an accumulative decision-making process and dopaminergic gain control—to explore how a more direct coupling between dopamine and decision-making may explain ramping dopamine signals in striatum.

#### Decision-making process

In the DDM, (differential) evidence *x*(*t*) is accumulated according to

$$\begin{array}{c} dx=Adt+cdW,\;x(0)=0, \end{array}$$ (17)

where the constant drift *A* = *Q*_1_ − *Q*_2_ represents the average increase in evidence supporting the correct choice per unit time (*Q*_1_ could represent the value of pressing a lever and *Q*_2_ the value of the null action—no lever press—for example), and *cdW* represents white noise which is Gaussian-distributed with *μ* = 0, *σ* ^2^ = *c* ^2^ *dt*. In the free-response case of interest here, the process terminates (i.e. decision is made) when *x* reaches a fixed threshold ±*z*. Analytic expressions relating error rate and decision times to DDM parameters can be derived in this simple case [73].

We consider the slightly augmented DDM in which the drift and diffusion (i.e. noise strength) constants vary over time [82]:

$$\begin{array}{c} dx=g(t)[Adt+cdW],\;x(0)=0, \end{array}$$ (18)

where *g*(*t*) is the time-varying gain which controls the drift and noise, and which we assume directly reflects dopamine concentration.

#### Dopamine dynamics

**Fig 10:**
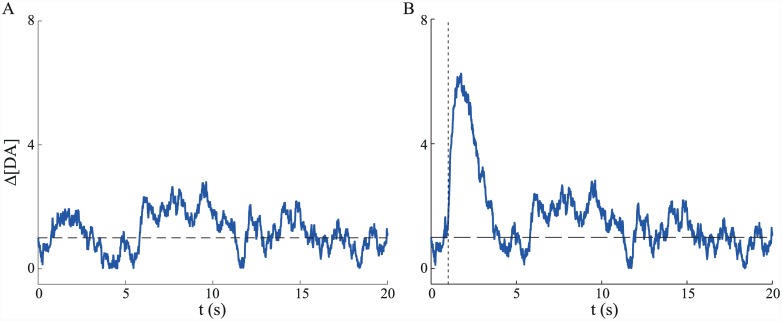
Simulated tonic and phasic dopamine fluctuations. (A) Simulated tonic fluctuations of dopamine concentration [DA] around a constant level (horizontal dashed line). (B) Addition of a comparatively large phasic fluctuation in dopamine concentration due to a TD error occurring at *t* = 1 s (vertical dashed line).

We consider the additive effects of two sorts of fluctuation in *g*(*t*): tonic and phasic. In the tonic case, dopamine is assumed to fluctuate in an autocorrelated manner around some constant level (Fig 10A). In particular, we assume that *g*(*t*) follows an Ornstein-Uhlenbeck process

$$\begin{array}{c} dg=\kappa (\theta -g)dt+\sigma dW,\;g(0)=\theta , \end{array}$$ (19)

where *θ* is the long-term mean of the process, *κ* controls the rate of mean reversion, and *σ* controls the variance of the white noise process.

In the phasic case, we consider the addition of a more dramatic change in dopamine concentration, notionally driven by TD-related phasic activity of dopamine cells occasioned either by an external cue or, as in the previous section, by a latent event internal to the animal (Fig 10B). Thus, tonic fluctuations are again assumed, as per Eq (19), but now a large phasic increase in dopamine is added to this signal. In particular, we assume phasic increases are driven by a TD response of random magnitude *h* drawn from a Gaussian distribution

$$\begin{array}{c} h∼N(\mu_{TD},\sigma_{TD}^{2}). \end{array}$$ (20)

As before, the TD response is converted to a transient change in extracellular dopamine concentration via the alpha function ‘DRF’ described in Eq (4).

### Tonic and phasic dopamine fluctuations produce average ramping signals

#### Tonic fluctuations

**Fig 11:**
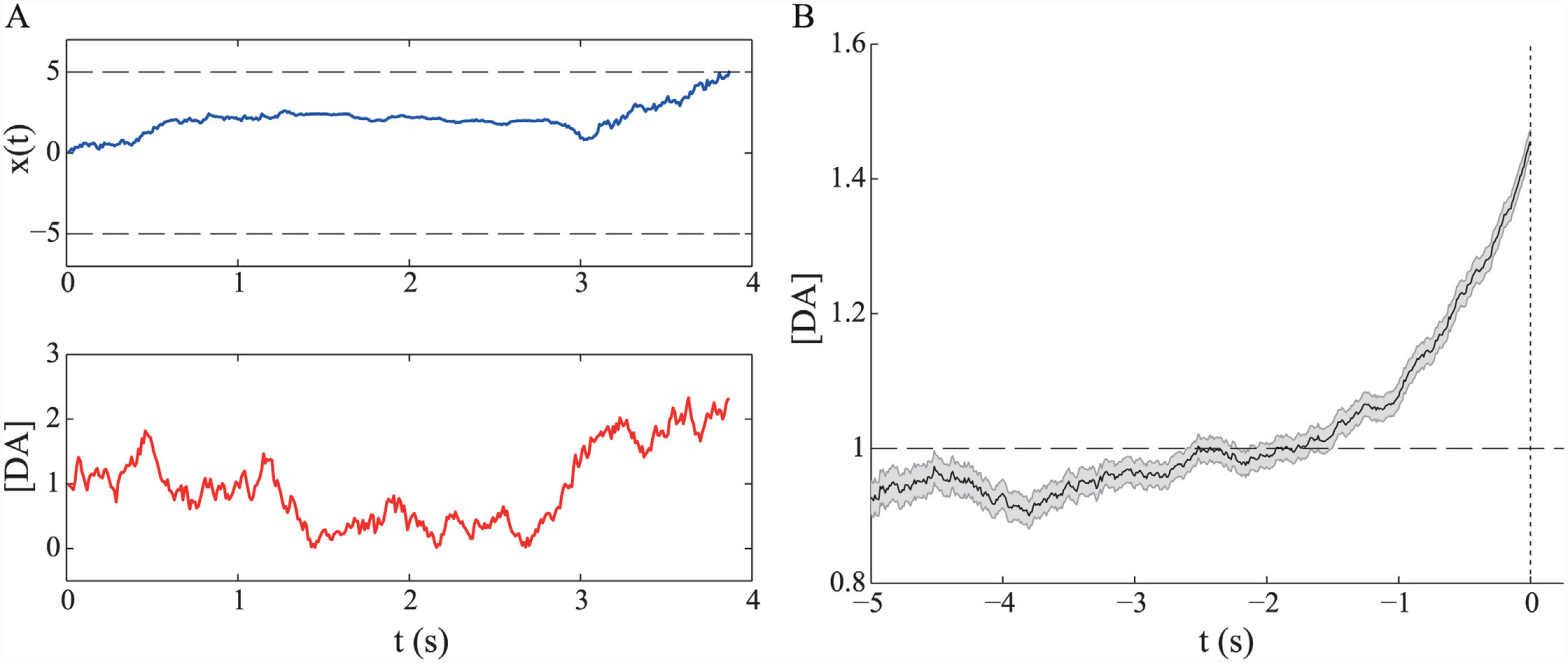
Tonic fluctuations generate average ramping signals. (A) Single trial example showing evolution of the decision variable *x*(*t*) (upper) and dopamine concentration [DA] (lower) over time. (B) Average [DA] (±SEM) aligned to time of threshold crossing. Number of simulated trials *N* = 1000. [DA] process parameters: *dt* = 0.01 s, *θ* = 1, *κ* = 0.01, *σ* = 0.1. DDM parameters: *A* = 1, *c* = 1, *z* = 5.

Fig 11A shows a single-trial example of how the decision-making variable *x*(*t*) and dopamine concentration *g*(*t*) co-evolve in the case of tonic dopamine fluctuations (upper and lower plots, respectively). Even though dopamine fluctuations here are driven purely by noise, averaging over dopamine signals aligned to the time of decision (i.e. threshold-crossing) reveals a clear ramping of this average signal towards decision time (Fig 11B). This averaging phenomenon is due to threshold-crossing events being more likely to occur when [DA] (i.e. the gain) is high, and also to the fact that the [DA] time series is autocorrelated.

#### Phasic fluctuations

**Fig 12:**
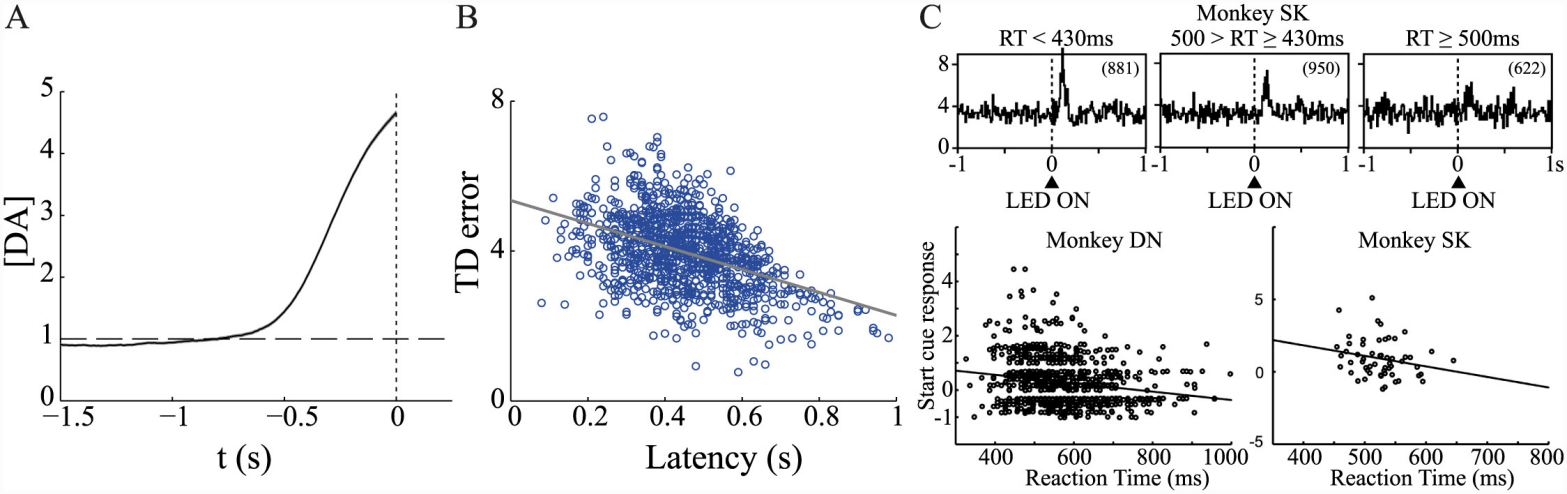
Phasic fluctuations generate ramping signals. (A) Average dopamine concentration [DA] (±SEM) aligned to time of threshold crossing. Number of simulated trials *N* = 1000. (B) Time of threshold-crossing (latency) is negatively correlated with size of TD error *h* in the model (*ρ* = −0.43). (C) Similarly, response magnitude of dopaminergic cells to a trial-start cue (upper plots, showing population response histograms by behavioural reaction time, RT) is negatively correlated with a monkey’s reaction time (lower) in an instrumental, reward-related task. Adapted from [83], with permission. [DA] process parameters: *dt* = 0.01 s, *θ* = 1, *κ* = 0.01, *σ* = 0.1. DDM parameters: *A* = 2, *c* = 0.1, *z* = 5. TD errors: $\mu_{TD}=4,\sigma_{TD}^{2}=1$.

Unsurprisingly, the addition of strong phasic fluctuations, notionally driven by TD-related activity, also generates an average ramping signal (Fig 12A). Of note in this case is the negative correlation between the magnitude of the TD response *h* and latency (i.e. time of threshold crossing; Fig 12B). This is in accord with the finding that the size of phasic responses of dopaminergic cells to a trial-start cue in a reward-related task is negatively correlated with reaction time [83] (Fig 12C).

#### Dopamine fluctuations and motivational state

**Fig 13:**
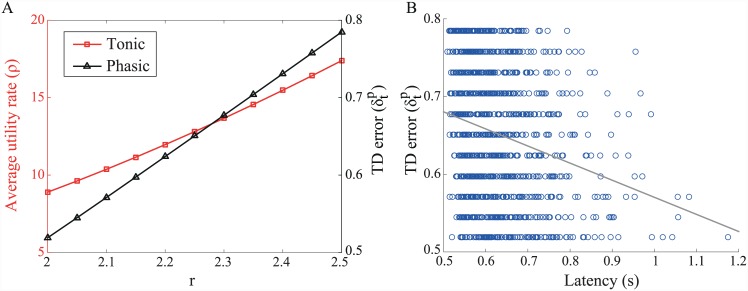
Correlation of average utility rate and size of TD error. (A) As the utility *r* of a reward increases, putatively from a change in motivational state, both the average utility rate *ρ* (assumed to be signalled by tonic dopamine) and the size of TD error $\delta_{t}^{p}$ in response to a trial-start cue (phasic dopamine) increase. (B) A negative correlation between TD error and latency is observed. Here, we again assume the lever-pressing task depicted in Fig 3B. The critic is assumed to receive only indirect information about the actor’s choices. Model parameters: *a* = −0.05, *b* = 0, *τ*_*post*_ = 0.5 s, *τ*_*I*_ = 0 s, *β* = 1.

While an obvious source of phasic fluctuations in dopamine concentration is the TD-related phasic activity of dopamine cells, the origins of the tonic fluctuations assumed here are perhaps less clear. Although these could simply be attributed to ‘intrinsic noise’, a psychologically richer possibility is that such fluctuations could be at least partially driven by changing motivational states. Indeed, Satoh et al. [83] suggested that the relationship between phasic DA activity and response latency that they observed was driven by changes in motivation. Returning briefly to the average-reward RL framework, we can consider what to expect in terms of dopaminergic activity and response latencies in different motivational states. Taking the lever-pressing case of Fig 3B, and a critic that only receives indirect information about the actor’s choice of *τ*, we now assume that the utility of a fixed reward depends on motivational state. Further, it is assumed that the actor selects a latency *τ* with a probability which depends on its relative Q-value via the softmax function: $P\left(\tau \right)\propto \exp(\beta {\tilde{Q}}^{\pi }\left(1,\tau \right))$, *β* = 1. In this case, it is straightforward to show that the average utility rate and magnitude of prediction error in response to a trial-start cue will be positively correlated (Fig 13A), either predictively given model-based calculations, or through experience of model-free ones [84]. Furthermore, it will again be the case that latencies are anticorrelated with the magnitude of prediction errors (Fig 13B). This example not only illustrates a possible role for motivational state in driving changes in dopamine levels and correlated changes in behaviour, but flags the difficulty of disentangling the influences of phasic and tonic dopamine on behaviour. Indeed, within the particular implementation of the average-reward RL model suggested by Niv et al. [39], the observed effect on latency is really determined by the tonic level of dopamine, which, in turn, is treated as being mechanistically independent of phasic dopamine. However, since changes in tonic level are correlated with changes in phasic response, what amounts to a spurious correlation between phasic activity and latency is observed.

### Ramping as state prediction

We now consider a third account of dopamine ramps based on a new model of discounted vigour. Incorporating the observations and suggestions of Howe et al. [14], together with a partially free-operant experiment of his own, Berke and colleagues (personal communication, [85]) suggested that the concentration of dopamine measured by FSCV in the accumbens might be strongly influenced by the discounted value function *V*(*s*) of Eq (1). This will show evidence of ramping towards final goal states when the discount factor is less than 1, consistent with the observations of Howe et al. (Fig 5). We describe this signal as being *quasi-tonic* since, when there is no reward, it is a form of integral of the TD prediction error, which is phasic. However, one should bear in mind that when the state changes abruptly, the value can change abruptly too. The key question, though, is why we should expect to see any such quasi-tonic signal in this context?

We consider the possibility that this signal is the equivalent in the discounted case of the average reward *ρ* (for convenience, in this section, we omit the superscript *π*) which, as we have seen, has previously been argued to be (a) the comparison point for the phasic prediction error or the immediate reward; (b) the spur to instrumental vigour; and (c) represented by tonic levels of dopamine [39].

Indeed, consider afresh an apparent inconsistency in the definition of the TD prediction error between the cases of average and discounted reward. In the average case, the phasic component of the full prediction error (c.f. Eq (16)), now denoted *δ* ^*A*^(*s*_*t*_), is

$$\delta^{A}\left(s_{t}\right)=r\left(s_{t}\right)+\tilde{V}\left(s_{t+1}\right)-\tilde{V}\left(s_{t}\right),$$ (21)

and we expect the mean of this over the long run to be the overall mean reward rate

$$\langle \delta^{A}\left(s_{t}\right)\rangle =\rho ,$$ (22)

which is a tonic signal that therefore acts as a comparison point for the phasic prediction error. Eqs (21) and (22) can also be seen as arising from the observation that the relative values $\tilde{V}$ are expected undiscounted sums of the differences between *r*(*s*_*t*_) and *ρ*. Unfortunately, even if the relationship in Eq (22) actually holds, *ρ*, because it is stationary, is formally hard to measure with FSCV, whose measurements are typically referenced to a potentially ever-changing baseline.

By contrast, in the discounted case, the phasic prediction error *δ* ^*γ*^(*s*_*t*_) is normally written as

$$\delta^{\gamma }\left(s_{t}\right)=r\left(s_{t}\right)+\gamma V^{\gamma }\left(s_{t+1}\right)-V^{\gamma }\left(s_{t}\right),$$ (23)

now writing the discounted value function as *V* ^*γ*^(*s*), and is expected on average to be 0:

$$\langle \delta^{\gamma }\left(s_{t}\right)\rangle =0.$$

However, two considerations encourage us to write this expression slightly differently, with an *undiscounted* phasic TD prediction error just as in Eq (21):

$$\delta^{A_{\gamma }}\left(s_{t}\right)=r\left(s_{t}\right)+V^{\gamma }\left(s_{t+1}\right)-V^{\gamma }\left(s_{t}\right),$$ (24)

which should, on average, take the value

$$\langle \delta^{A_{\gamma }}\left(s_{t}\right)\rangle =\left(1-\gamma \right)\langle V^{\gamma }\left(s_{t+1}\right)\rangle .$$ (25)

Here, (1 − *γ*)〈*V* ^*γ*^(*s*_*t* + 1_)〉, by analogy with the truly stationary signal *ρ*, would be represented as a quasi-tonic signal which acts as a target for a phasic TD prediction error signal that involves a discounted value function. Assuming that this baseline signal is represented in a quasi-tonic concentration signal would thus licence ramping.

The two considerations that encourage this interpretation of phasic and quasi-tonic dopamine signals are: (i) continuity between average and discounted cases as *γ* → 1; (ii) something of particular pertinence in the current context, namely the determinants of vigour for discounted problems. We discuss these in turn. There is also a rough analogy with the Hamilton-Jacobi-Bellman (HJB) equation [86, 87], but as this requires considering continuous space and time, as well as a different sort of transition structure, we do not discuss it further.

#### Continuity

It is well known that, in convenient circumstances, there are very close links between the infinite horizon average and discounted reward cases for dynamic programming and control [42, 86]. For instance, for a large class of suitable MDPs, there is a minimum discount factor 0 ≤ γ* < 1 such that the optimal policy for the discounted problem with discount *γ* > γ* is also optimal for the average case. Thus, we might expect the prediction errors and putative phasic and tonic signals to be continuous as *γ* → 1. This is patently not true of the single Eq (23), compared with the pair Eqs (21 and 22).

However, it is well known that this can be repaired by considering the term in Eq (25) in the limit that *γ* → 1:

limγ→1(1-γ)Vγ(s)=limγ→1limN→∞∑k=0N-1γkr(sk)s0=slimN→∞∑k=0N-1γk,

where the denominator arises from the fact that 1+y+y2+…=1(1−y),

=limγ→1limN→∞∑k=0N-1γkr(sk)s0=s∑k=0N-1γk.

Thus, if the limits can be swapped (see [86] for conditions),

=limN→∞limγ→1∑k=0N-1γkr(sk)s0=s∑k=0N-1γk=limN→∞1N∑k=0N-1r(sk)s0=s=ρ,

and so a formal continuity as *γ* → 1 does arise between the two pairs of phasic and tonic representations Eqs (21 and 22) and Eqs (24 and 25).

#### Discounted vigour

As mentioned above, one of the main suggestions about the tonic release of dopamine associated with *ρ* is that it should determine the vigour of responding. This is shown in the average reward model discussed above by the influence of this factor in the latency of Eq (10) that maximises the relative value $\tilde{Q}$ of Eq (9).

In the discounted case, the equivalent *Q* ^*γ*^ equation to Eq (9) is

$$\begin{array}{cc} Q^{\gamma }\left(s_{t},\tau \right) & =c\left(\tau \right)+r\left(s_{t}\right)+\gamma^{\tau }V^{\gamma }\left(s_{t+\tau }\right)\\ & =c\left(\tau \right)+r\left(s_{t}\right)+V^{\gamma }\left(s_{t+\tau }\right)-\left(1-\gamma^{\tau }\right)V^{\gamma }\left(s_{t+\tau }\right), \end{array}$$

showing that the opportunity cost −*ρτ* encouraging quick actions has been replaced by the ever more negative value of −(1 − *γ* ^*τ*^)*V* ^*γ*^(*s*_*t* + *τ*_) as *τ* increases. As *γ* → 1, (1 − *γ* ^*τ*^) can usefully be written as *τ*(1 − *γ*) + *O*((1 − *γ*)^2^), so the portion of *Q* ^*γ*^(*s*, *τ*) that depends on *τ* becomes

$$\begin{array}{cc} Q^{\gamma }\left(s,\tau \right) & =K+c\left(\tau \right)+\gamma^{\tau }V^{\gamma }\left(s_{t+\tau }\right)\\ & ≃K+c\left(\tau \right)-\tau \left(1-\gamma \right)V^{\gamma }\left(s_{t+\tau }\right), \end{array}$$

and a comparison with Eq (9) shows again how (1 − *γ*)*V* ^*γ*^(*s*_*t* + *τ*_) in the discounted reward model plays an equivalent role to *ρ* in the average model. The equations above extend this to the non-limiting case of *γ* < 1.

### A quasi-tonic dopamine signal

**Fig 14:**
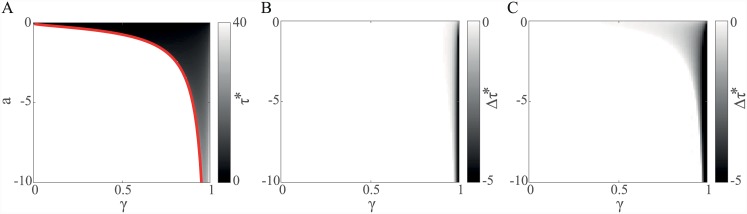
Optimal latency τ* as a function of discount factor *γ* and cost *a*. The optimal latency τ* tends to decrease as either the discount factor *γ* or the cost of acting quickly *a* decrease. (A) Terminating SMDP, *V* ^*γ*^(*s*_*t* + *τ*_) = 1, ∀*τ*, *γ*. There exists a limit on the cost of acting *a*_lim_ below which there is no solution for τ* (solid red line). (B) Difference between the optimal τ* for the cases of continuing and terminating SMDP for the case that *τ*_*I*_ = 30 s. As *τ*_*I*_ is large relative to −1/log *γ*, there is little difference Δτ* from the terminating case. (C) Difference between the optimal τ* for the cases of continuing and terminating SMDP for the case that *τ*_*I*_ = 1 s. In this case, future rewards hasten lever pressing as seen in the more prevalent decreases in τ*.

Insight into discounted vigour comes from numerical calculations of the optimal latencies τ* as a function of *γ* and for different values *a* < 0 that control the hyperbolic cost of vigour $c\left(\tau \right)=\frac{a}{\tau }$ in two cases: a terminating chain with *V* ^*γ*^(*s*_*t* + *τ*_) = 1, ∀*τ*, *γ* (roughly as in [14]) and a continuing chain as in Fig 3. Fig 14A shows optimal latencies τ* in the terminating case. Generally, as *γ* decreases, the faster the weight given to future value *V* ^*γ*^(*s*_*t* + *τ*_) decays with time, encouraging quicker latencies. This tendency is balanced by the greater cost of acting quickly that is then incurred. In fact, one can show that there there is a limit on the cost of acting of *a*_min_ = 4/(*e* ^2^ log *γ*) below which there is no solution for τ* — crudely, the cost of acting quickly deems such a long latency that the resulting discounted value of the reward (from *V* ^*γ*^(*s*_*t* + *τ*_) = 1) is insufficient to warrant action at all (Fig 14A, solid red line).

Fig 14B and 14C show *differences* in τ* in the continuing compared to the terminating case. In the continuing SMDP, the result of pressing the lever includes a further opportunity to press the lever (without which, the infinite horizon average reward *ρ* would formally be 0). When the inter-trial interval *τ*_*I*_ is large relative to −1/log *γ*, there is little difference from the terminating case (Fig 14B); however, when it is not, the prospect of accelerating not only the immediate reward but also future rewards further hastens lever pressing, visible in greater decreases in τ* compared to the terminating case (Fig 14C).

**Fig 15:**
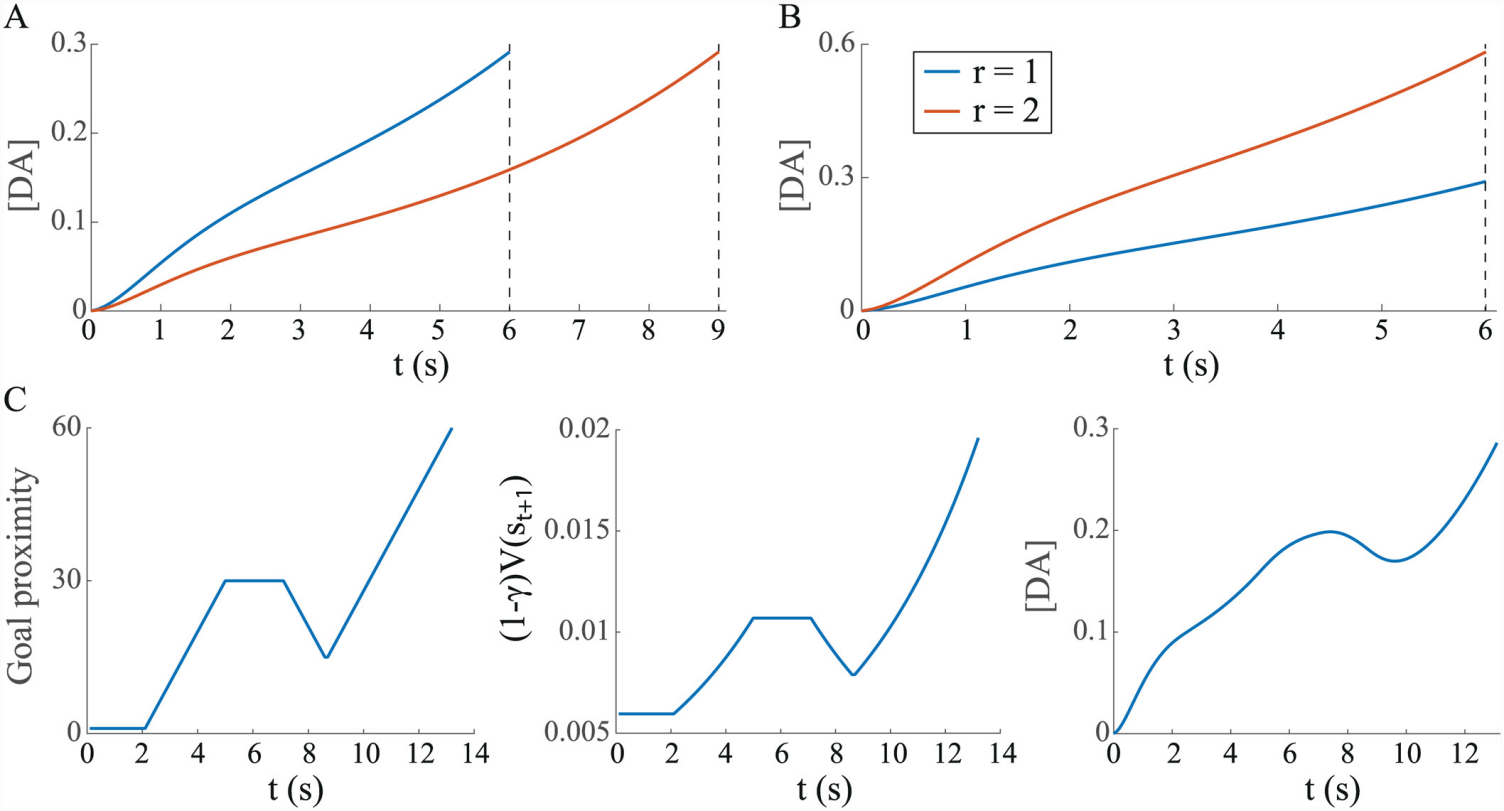
Simulations replicate Howe et al. results. (A) [DA] gradually increases as the goal is approached, peaking at the same values whether different times were taken to traverse a maze of fixed length, or in mazes of different lengths with a fixed magnitude of reward (time is taken as a proxy for distance in the latter case). (B) Peak [DA] is greater for larger rewards. (C) [DA] tracks proximity to the goal. In this example, goal proximity over time is non-monotonically increasing (left), and we plot both the corresponding scaled value quantity (1 − *γ*)*V* ^*γ*^(*s*_*t* + 1_) (middle) and the convolution of the latter with the DRF which yields [DA] (right). Parameters: *γ* = 0.98, *r* = 1 (unless indicated otherwise).

Given the preceding analysis, it is straightforward to show that a quasi-tonic dopamine signal reflecting the quantity (1 − *γ*)*V* ^*γ*^(*s*_*t* + 1_) would lead to the sort of ramping observed by Howe et al. [14] in their spatial reward task (c.f. Fig 5) for *γ* < 1. Indeed, just as observed by Howe et al., [DA] gradually ramps up as the goal is approached and peaks at the same value regardless of the time taken to reach the goal or the distance travelled to reach it, assuming a fixed reward size (Fig 15A). Further, as observed experimentally, increasing the reward size leads peak [DA] to increase (Fig 15B) and, given a lack of progress towards the goal—for instance if the agent remains stationary or moves away from the goal—[DA] remains approximately stationary or decreases, respectively, as observed by Howe et al. on such trials (Fig 15C). One should note in this latter case that the single-trial examples shown by Howe et al. find dopamine concentrations tracking spatial proximity remarkably closely (see Fig 5C), while convolution of (1 − *γ*)*V* ^*γ*^(*s*_*t* + 1_) with the DRF that we have assumed leads to a signal which looks comparatively over-smoothed (Fig 15C, right). However, given the heterogeneous nature of striatal dopamine release [88], how rapidly [DA] is observed to change may well depend on the exact positioning of the voltammetric sensor. Examination of further single-trial examples could help clarify this issue.

## Discussion

The observation of ramp-like increases in dopamine concentration within the nucleus accumbens appears to pose a challenge to existing computational accounts of dopamine’s role. Here, we explored three different explanations for such signals: (a) resolution of uncertainty about the timing of action within an actor-critic, leading to a prediction error shortly preceding the action itself; (b) positive correlations between the time of action and dopamine levels generated by dopaminergic gain control of the decision-making process; and (c) a quasi-tonic signal replacing the average reward in the exponentially discounted setting. These explanations, along with the possibility mentioned earlier that release from dopamine axons might be directly occasioned by a form of spillover from cortico-striatal activity, are by no means mutually exclusive. This prompts a need for experimental test.

Note that the various cases of ramps may be caused by different, or combined, mechanisms. Indeed, the possible explanations that we considered mainly in the context of pre-response transients, in which ramp-like signals are observed leading up to completion of an instrumental action, were somewhat distinct from the explanation offered for ramping in the spatial reward task, in which the subjects are already engaged in acting. Nevertheless, the account of discounted vigour suggested in the latter case should be relevant in all contexts where some degree of discounting is probable (i.e. *γ* < 1), such as in the temporally-extended tasks considered here, since it is in this case that the quantity (1 − *γ*)*V* ^*γ*^(*s*_*t* + 1_) should be visible as a ramping signal.

### TD accounts of pre-response dopamine signals

What TD accounts of pre-response dopamine signals predict depends on the assumptions made about the relationship between actor and critic. We considered three possibilities associated with different predictions of how a TD error occurring just prior to pressing, and the resulting change in dopamine concentration, should change as response latencies increase: remain constant, decrease, or increase.

The model in which the critic receives *both* direct and indirect information, but suffers from timing uncertainty, yielded results most consistent with the experimental data reported by Roitman et al. [10]. In particular, this case replicated the observation that peak dopamine concentration around time of pressing was larger than at time of cue for long latency trials. This result relied on the assumption that the critic’s uncertainty about the time of action increases with choices of longer press latencies. This is consistent with the finding, in the equivalent Pavlovian circumstance, that the responses of dopaminergic neurons to a cue predicting reward delivery after a long delay are smaller than responses to cues predicting shorter delays; conversely, dopamine responses to the reward itself increase with longer delays [62, 89], a finding that indeed has been suggested to arise through timing uncertainty.

This finding is apparently opposite, though, to an observation also mentioned above. This is that for the case of a single, non-exponential hazard function, which mandates a range of possible times at which a reward-related cue might be presented, relatively late presentations inspire *smaller* dopamine responses than early ones [63]. An obvious explanation of this finding is that as time goes by, presentation of the cue is more and more likely, and so less and less unexpected. This does not contradict our finding, which depends on many possible hazard functions, one for each choice of lever-press latency.

Two assumptions in the proposed TD account merit further comment. Firstly, while we assumed that the actor’s choice of when to press the lever immediately follows cue presentation, one can imagine variability in when the actor makes decisions about when to act. For example, it might be that the animal initially fails to notice the cue, or is otherwise engaged (even in instrumental leisure; [40]) when the cue arrives, only later resolving to engage with the lever. Secondly, and perhaps relatedly, while it was convenient to assume that latencies *τ*, and therefore times *T*, follow a gamma distribution, the reported distribution of press times appears to have heavier tails than we would expect if they were drawn from a single gamma distribution. Thus, Roitman et al. reported mean response times of 1.2 s and 26.2 s for short-latency (83%) and long-latency (17%) trials, respectively. Closer examination of the empirical response distribution in such studies would be of interest for future work.

A more general problem for a TD account of pre-response signals is that while there is abundant evidence of a systematic temporal relationship between the time at which a cue indicating reward availability is presented and subsequent phasic activity in dopaminergic neurons, there is little or no evidence of such a relationship between the time of the phasic response and when a subsequent instrumental action—necessary to obtain the reward—is emitted. For example, Ljungberg et al. [90] found that when monkeys were exposed to cues that predicted when they could obtain food by reaching into a box, activity of dopaminergic neurons was time-locked to the predictive cue rather than movement onset. Whether this is also true for the timescale and nature of rodent movements is unclear. Even in monkeys, Romo and Schultz [91] reported gradual increases in the firing rates of some putative dopaminergic cells (12 out of 104 recorded) up to 1500 before onset of self-initiated arm movements to obtain food. However, this slow change in activity does not resemble the sort of bursting activity that might be associated with a phasic TD signal.

Suggestively, striatal (and cortical) neurons in monkeys show various patterns, including ramp-like increases in activity, before self-initiated movements [55, 92, 93]. Similarly, some neurons in rat ventral striatum show anticipatory increases in activity when approaching or waiting for food delivery [94, 95]. Furthermore, simultaneous electrophysiological and FSCV recordings from the same electrode have revealed that changes in dopamine concentration and activity of specific subsets of accumbal cells can be temporally correlated [96, 97]. Suppression of phasic activity in VTA dopaminergic cells appears to disrupt such time-locked activity, perhaps indicating that it is phasic activity of dopaminergic cells which drives such correlated activity [98]. So at the mechanistic level at least, there are multiple possibilities for the origins of pre-response signals beyond phasic dopaminergic activity: they may reflect the sort of slow change in activity of dopaminergic neurons observed in [91], or they may reflect increased dopamine release instigated more directly by the activity of other cells, such as reflected in cortico-striatal inputs.

A range of previous work has considered the vagaries of the representation and processing of time. We noted that possible sources of uncertainty included partial observability of the actor’s choices in the case where the critic does not have direct access to this information, and possible timing uncertainty in the case where it does. Implications of partial observability for TD models of dopamine have been explored in previous work, notably by Daw and colleagues [48], though that did not address the possibility of partial observability arising between distinct internal agencies, nor the possible relevance to self-initiated action envisaged here. The same study and a number of others [48, 49, 99–101] have addressed the issue of the representation of time, and how this representation may influence timing uncertainty (see [102] for a recent review). The implications for TD (and indeed different models of discounting) of the possible distinction between the animal’s ‘internal’ time and the experimenter’s ‘conventional’ time have been worked out in detail by Nakahara and Kaveri [49]; we also considered the possibility of separate internal clocks for actor and critic. Additional complexity, which we leave to future work, arises from the putative connection between dopamine and the speed of an internal clock, as inferred, for example, from the effects of dopamine manipulations on behaviour in interval-timing tasks [102, 103].

### Dopaminergic gain control

We showed that ramping dopamine signals can be generated by a mechanistic decision-making model in which dopamine sets the gain of value-based accumulation. Furthermore, we saw that this direct coupling of dopamine to decision-making could generate a negative correlation between the size of TD error and decision time, consistent with the experimental observation that a larger phasic response of dopaminergic cells to a start cue is associated with a shorter latency of behavioural response [83].

This route to ramping signals is primarily statistical, arising from trial-averaging. On any individual trial, dopamine ramping towards the time of decision may or may not occur, though it is certainly more typical when dopamine fluctuations incorporate a strong, TD-related phasic component. To the best of our knowledge, whether pre-response transients in NAc reliably precede the animal’s response on individual trials, or may reflect trial-averaging, is unknown.

Mathematical analysis of the time-varying gain DDM that we described is given by Moehlis et al. [82] and, indeed, the idea that dopamine could set this gain follows directly from previous work by Cohen and colleagues on catecholaminergic gain control [79, 80]. For example, Shea-Brown et al. [80] suggested that noradrenergic activity of cells in the locus coeruleus may help to optimize decision-making by adjusting the gain of an integrative decision process. Furthermore, they showed that their model could replicate the experimental finding that phasic responses of the locus coeruleus correlate more closely with time of behavioural response than with time of stimulus onset in a decision-making task [104].

Also of relevance is the biologically-detailed neural network model of the basal ganglia proposed by Frank [81] in which dopamine modulates the balance between direct and indirect pathways. Ratcliff and Frank [105] have recently explored the links between the latter’s neural network model and more abstract diffusion models, though without exploring a possible direct role for dopamine in the latter. Nevertheless, it is interesting to consider that, depending on the form of the DDM used to fit the data, dopaminergic modulation of a temporally-extended decision process may be manifest in different parameters. For example, a positive correlation between increased tonic dopamine levels and faster responding may also be captured by the assumption that dopamine modulates the threshold of a DDM where the gain is fixed [106], rather than modulating the gain under a fixed threshold. Additionally, one may consider potential effects of dopamine not only on the latency of response, but also on which choice is made, for instance due to asymmetries in how dopamine modulates direct and indirect pathways (M.J. Frank, personal communication; [107]). More generally, it would be of interest to know whether dopamine ramps would also be observed in Frank’s comparatively detailed model of the basal ganglia.

### Discounted vigour

We reconciled an apparent inconsistency between the definitions of TD errors in the cases of average and discounted reward via an analysis in which ramp-like signals would be expected to emerge. In particular, we suggested that the quantity (1 − *γ*)〈*V* ^*γ*^(*s*_*t* + 1_)〉 in the discounted reward model plays an equivalent role to the average reward rate *ρ* in the average reward model. Since values often (though not always) change modestly as a result of the passage of time, this signal is quasi-tonic, and thus a candidate for what would be recorded using a technique such as FSCV. This signal can explain the ramping phenomena observed by Howe et al. [14] and also those observed in more recent experimental work [85]. We speculate below on its network or biophysical realization.

Potentially at odds with our suggestion that the quantity (1 − *γ*)〈*V* ^*γ*^(*s*_*t* + 1_)〉 is appropriate for controlling vigour, changes in the running speed of rats in Howe et al.’s study do not show a close match to the temporal profile of dopamine concentrations. However, one would not necessarily expect a straightforward relationship between these variables, given that the subjects must negotiate environments without crashing into walls. Howe et al. used T-, M-, and S-shaped mazes, whose turns, unsurprisingly, led to decreases in velocity (see [14], figure 3h–k).

Our analysis suggests that ramps are scaled by the discount factor *γ*, prompting the question of how this discount factor is set, whether it is variable or fixed, and indeed, whether it is unique. There is substantial evidence that human and animal discounting takes a hyperbolic form [108, 109] rather than being exponential as considered here (which is rather ubiquitous in engineering and economic settings). This can arise from a combination of two or more exponentials, and it would be most interesting to extend our analysis to this case. From a formal viewpoint, the discount factor can be seen as the probability per unit time of task termination or, indeed, as a means of simplifying a problem en route to an ultimate solution [110].

In humans, there is evidence that discount rates can be manipulated experimentally [111] and that individuals can flexibly vary their discount rates to suit task demands [112]. It has also been suggested that some regions, notably the striatum, display a graded map of discount rates which serve reward prediction at different timescales [113, 114]. Howe et al. observed ramping in dopamine concentration in both ventromedial and dorsolateral striatal areas, though ramping responses were reported to be more common in ventromedial striatum. Hints of steeper ramping are perhaps discernible in the average signals reported in ventromedial as opposed to dorsolateral striatum ([14], figure 1 and extended data figures 3a and 4). However, whether such ramping signals display systematic, graded differences across the striatum or otherwise change in response to experimental manipulation of discount factors remains an open question.

### Complexities of dopamine release: Phasic, tonic, and quasi-tonic

Whereas the TD account of pre-response transients naturally attributes the observed signal to the phasic activity of dopaminergic neurons [2, 5–7, 19], the sources of tonic and particularly ‘quasi-tonic’ dopamine signals are less clear. One long-standing suggestion is that phasic and tonic modes of firing in dopaminergic cells provide independent control of phasic and tonic dopamine levels within NAc [26, 115, 116]. Thus, burst firing of dopaminergic neurons is thought to mediate a fast, high-amplitude dopamine transient which is spatially-restricted to a region within or proximal to release terminals by dopamine reuptake. By contrast, the comparatively slow, irregular, ‘tonic’ mode of activity exhibited by a pool of dopaminergic neurons, potentially of varying size, is thought to control the more stable, tonic levels of extrasynaptic dopamine. If average reward rate is represented in tonic levels of dopamine [39], then a natural suggestion is that representation of this quantity is controlled by this tonic mode of activity.

Where does a quasi-tonic dopamine signal fit into this picture? It is not clear that the relatively short timescale of change of the ramping signals reported by Howe et al. could arise through mechanisms thought to modulate tonic activity. On the other hand, ramping in the phasic activity of dopaminergic neurons has seldom been reported. Fiorillo et al. [117] reported ramp-like increases in between-trial averaged activity under conditions of uncertain reward delivery, though interpretation of this result has been controversial [25, 118]. While the paucity of such reports may simply be due to a lack of appropriate electrophysiological recordings in spatial tasks—which may also explain why ramping of dopamine concentrations has not been observed prior to [14]—an interesting alternative is that the gradual increase in dopamine concentration is partially- or fully- independent of the activity of dopaminergic cells [15]. As mentioned above, a number of local regulatory mechanisms are known to gate the probability of dopamine release [27, 28], and there is evidence that striatal dopamine release can occur independently of dopamine cell firing [119]. An understanding of how these different mechanisms of dopamine release interrelate is of clear experimental and theoretical interest.

It should be noted that although we have referred throughout to dopamine signals in the nucleus accumbens generally, this should not be taken to suggest that dopamine release is homogeneous within this region. Indeed, FSCV measurements suggest substantial spatial heterogeneity [88]. Subregions of NAc have been segregated according to various anatomical features, classically into core and shell subregions [120, 121]. Pre-response transients have typically been observed in NAc core [8–13]. Much interest centres on the functional significance of this core-shell distinction [37, 122–125] and, indeed, distinctions at a finer grain [126], including in relation to possible differences in dopaminergic release [127].

### Alternative accounts

We noted above that ramping ostensibly disrupts TD’s explanation for dopaminergic release, since it would have, oxymoronically, to be a predictable prediction error. Alternative accounts have been suggested according to which prediction errors indeed persist.

Gershman [128] considered the consequences of an unsuitable state representation. The idea is that the exponentially discounted value signal *V* ^*γ*^(*s*) cannot be captured in an error-free manner if the state (i.e., the position of the animal) is represented in particular, over-generalizing manners, for instance by units whose activity is governed by the square, rather than linear, distance to the goal. In this case, a ramping prediction error turns out to arise via persistent representational error. Place cells [129–131] provide an accessibility-sensitive representation of space, and the generalization afforded by the coarse-coding they imply is often useful [132]. However, it is also known that Bayesian decoding of even a modest number of such cells leads to surprisingly accurate localization of animals in their environments [133], and thus what would amount to a table-lookup representation that would not lead to persistent error. Of course, one must remember that this sort of decoding is *in silico*, rather than *in vivo*.

Morito and Kato [134] have also also suggested that the Howe et al. ramping signal reflects persistent prediction errors. In their proposal, these arise out of the assumption of a time-dependent decay of learned state values. One challenge for this model is that its generation of ramping signals qualitatively similar to that observed experimentally appears to be unstable to changes in reward magnitude [134], and indeed to the passage of more substantial periods of time.

### Experimental tests

The most pressing consideration is a set of experiments that can test and refine or reject these various mechanisms, and understand how they might work together. Perhaps the most straightforward to test is the last suggestion, since it is unique in its dependence on discounting. Given that the rate of this should be sensitive to things like the reliability of the environment [135], it would be interesting to manipulate these factors, determine the extent to which behaviour changes appropriately, and concurrently measure ramping. Similarly, it may be that individual differences in discounting, as measured by choices between immediate, smaller rewards and delayed, larger rewards, can be predicted by the rate of ramping. Although behaviour generally follows hyperbolic rather than exponential discounting [109], this would only make a modest difference at the timescales that appear relevant for the sort of ramping behaviour observed by Howe et al.

Testing the second suggestion could be accomplished using photo-uncaging of dopamine in the accumbens (for instance, using RuBi-Dopa [136]), since of the three mechanisms, it suggests the strongest coupling between dopamine and immediate behaviour. Optogenetically-stimulated release (using TH-CRE or DAT-CRE lines) could also be employed, although it would then be hard to distinguish the specifically dopaminergic component from any other influences of the (potentially antidromically-stimulated) activity of the dopamine neurons. It would be interesting to contrast the results of this with direct stimulation of D1-receptor-containing and D2-receptor-containing neurons [137] to try to assess downstream mechanisms.

Testing the relationship between actor and critic is particularly tricky, since we know so little about the implementation (or indeed existence) of either and, in particular, the micro- or nano-scopic nature of choice over time [40]. Nevertheless, it would certainly be interesting to compare the nature and magnitude of ramping when subjects are made to wait for shorter or longer times, with and without cues for the precise passage of time that could be exploited.

More generally, key issues surround the relationships between the number of dopamine cells that are active, the phasic and tonic activity of those neurons, the spatiotemporal profile of the concentration of dopamine at receptor targets in the accumbens, and the action of this dopamine on those receptors (along with the action on target neurons of other neurotransmitters co-released by the same neuronal activity). This information is key for making qualitative and ultimately quantitative progress.
